# Interim opioid agonist treatment for opioid addiction: a systematic review

**DOI:** 10.1186/s12954-022-00592-x

**Published:** 2022-01-29

**Authors:** Laura Samsó Jofra, Teresa Puig, Ivan Solà, Joan Trujols

**Affiliations:** 1 Department of Epidemiology and Public Health, Hospital de la Santa Creu i Sant Pau, Barcelona, Spain; 2 Biomedical Research Institute Sant Pau (IIB Sant Pau), Barcelona, Spain; 3 Facultat de Medicina, Universitat Autònoma de Barcelona (UAB), Cerdanyola del Vallès, Spain; 4 CIBER Enfermedades Cardiovasculares (CIBERCV), Instituto de Salud Carlos III (ISCIII), Madrid, Spain; 5 CIBER Epidemiología y Salud Pública (CIBERESP), Instituto de Salud Carlos III (ISCIII), Madrid, Spain; 6 Iberoamerican Cochrane Center, Barcelona, Spain; 7 Unitat de Conductes Addictives, Servei de Psiquiatria, Hospital de la Santa Creu i Sant Pau, Sant Antoni Maria Claret 167, 08025 Barcelona, Spain; 8 CIBER Salud Mental (CIBERSAM), Instituto de Salud Carlos III (ISCIII), Madrid, Spain

**Keywords:** Opioid use disorder, Opioid agonist treatment, Interim treatment, Methadone, Buprenorphine, Systematic review

## Abstract

**Background:**

Opioid use disorder is a public health problem and treatment variability, coverage and accessibility poses some challenges. The study’s objective is to review the impact of interim opioid agonist treatment (OAT), a short-term approach for patients awaiting standard OAT, in terms of treatment retention, access to standard OAT, quality of life and satisfaction with treatment.

**Method:**

We conducted a systematic review searching MEDLINE, EMBASE, PsycINFO, and CENTRAL up to May 2020. Due to variability between studies and outcome measurements, we did not pool effect estimates and reported a narrative synthesis of findings rating their certainty according to GRADE.

**Results:**

We identified 266 unique records and included five randomized trials with some limitations in risk of bias and one observational study limited by selection bias. The studies assessed similar approaches to interim OAT but were compared to three different control conditions. Four studies reported on treatment retention at 4 months or less with no significant differences between interim OAT and waiting list or standard OAT. Two studies reported treatment retention at 12 months with no differences between interim OAT and standard OAT. Two trials assessed access to standard OAT and showed significant differences between interim OAT and waiting list for standard OAT. We rated the quality of evidence for these outcomes as moderate due to the impact of risk of bias. Data on quality of life or satisfaction with treatment was suboptimal.

**Conclusions:**

Interim OAT is likely more effective than a waiting list for standard OAT in access to treatment, and it is probably as effective as standard OAT regarding treatment retention.

*PROSPERO registration* CRD42018116269.

**Supplementary Information:**

The online version contains supplementary material available at 10.1186/s12954-022-00592-x.

## Background

Opioid agonist treatments (OATs) reduce injection-related mortality and morbidity [1–3], all-cause and overdose mortality [4–6], and improve quality of life [7, 8] among people with opioid use disorder. In fact, the safety, efficacy and effectiveness of the two most commonly used OAT medications (i.e., methadone and buprenorphine) have been widely researched and proved [9–12].

Despite this evidence, there is great worldwide variability in OAT practices and coverage. A recently published, global systematic review [13] found wide variation between—and within—countries in the way OAT is delivered (e.g., treatment eligibility criteria, mean opioid dose prescribed, access to unsupervised dosing, and urine drug screening practices) in routine clinical practice.

By 2020, many countries in Asia, Latin America and Africa had very low levels of OAT coverage [14]. In Europe it is estimated that treatment coverage is around 50% but there is also great variability and some countries have low or insufficient levels (< 30%) [15]. In USA, demand for OAT far exceeds available capability, with an alarming number of OAT clinics having extensive waitlists (even of years) [16] and, despite the current opioid crisis, treatment coverage does not improve proportionally [17]. These waitlists have a direct detrimental effect on people awaiting treatment, placing them at high risk for criminal activity, infectious disease, overdose, and mortality [16, 18]. In fact, the costs of untreated opioid use disorder of patients placed on a methadone treatment waiting list also entail a significant financial burden to society [19].

In this context of insufficient coverage and increasing waiting lists, low-threshold treatments such as interim OAT have been proposed and introduced. This treatment option provides limited services to people with opioid use disorder who would otherwise be on a waiting list for comprehensive, standard OAT. Interim clinics usually provide intake physical examination, education about acquired immune deficiency syndrome (AIDS) and opioid agonist medication but do not provide psychosocial interventions as such. They are expected to bridge waitlist for at risk populations and reduce harm to them; therefore, these clinics’ main aim is harm reduction. Interim OAT is dispensed daily by a nurse and usually taken with direct observation, and the treatment should last no longer than 120 days [20]. Interim OAT was first mentioned in the 1970’s [21] and has been introduced in some countries since then. In USA, it was approved by the Food and Drug Administration (FDA) in 1993 [22].

Given the current opioid crisis, the ongoing global variability in OAT’s accessibility and coverage keeps putting patients at risk and has negative consequences for both patients and society. Interim OAT has been proposed as an option to mitigate these problems but has not yet been evaluated globally.

The objective of the study is to conduct a systematic review in order to synthetize the current knowledge on the following clinical question (according to the PICO framework): among people with opioid use disorder (population), is interim OAT (intervention), compared to other approaches (comparison), more effective and cost-effective in terms of retention in treatment, access to standard OAT, quality of life or well-being, satisfaction with treatment, use of non-prescribed psychoactive substances, criminal activities, mental and physical health status and adverse effects (outcomes)?

## Method

We conducted a systematic review according to a protocol (International Prospective Register of Systematic Reviews (PROSPERO) registration number CRD42018116269), which is available at Open Science Framework (https://osf.io/fsvte/), following the methodological standards from Cochrane Collaboration [23] and reported the results following the Preferred Reporting Items for Systematic Review and Meta-Analysis (PRISMA) statement [24] (Additional file 1).

### Study selection

We included studies that: (1) assessed interim methadone or buprenorphine treatment for individuals with opioid use disorder awaiting entry into standard OAT; (2) included any control condition such as placebo, no intervention (waiting list), or standard OAT; (3) were published in a peer-reviewed journal. In addition to randomized control trials we included comparative observational studies, follow-up studies assessing the long-term effects of interim OAT, and economic evaluation studies.

Eligible studies included patients with an opioid use disorder diagnosed according to standardized criteria (e.g., Diagnostic and Statistical Manual of Mental Disorders (DSM), International Classification of Diseases (ICD)) who were candidates to be admitted into a standard OAT. We included studies assessing an interim OAT (methadone or buprenorphine) defined by the provision of opioid agonist medication without other interventions to people who were suffering opioid use disorder and whose only other option would have been a waiting list for the standard OAT [22].

Given (i) the anticipated heterogeneity of outcome variables in eligible studies, (ii) the lack of a core outcome set for OAT research [25–27], and (iii) the fact that some of the commonly used outcome variables in OAT evaluations may not adequately reflect patient perspectives [28–30], we decided to not restrict our review to a few particular outcome variables. We considered the following primary outcomes: retention in treatment, access to standard OAT, quality of life or well-being and users’ satisfaction with treatment. Secondary outcomes were the use of illicit opioid and/or non-prescribed psychoactive substances, criminal activity and/or illegal income, mental and/or physical health status, the rate of adverse effects and the costs of this intervention.

### Search methods for identification of studies

We searched MEDLINE, EMBASE, PsycINFO, and Cochrane Central Register of Controlled Trials from their inception until May 2020, without limitations in language or publication status. We designed a search strategy (Additional file 2) using text words related to the intervention and population of interest combined with controlled vocabulary, adapted to the requirements of each database. We searched the International Clinical Trials Register for ongoing studies. Additionally, we screened the reference lists of relevant studies.

### Data extraction and management

We constructed a database in a reference management software (EndNote X2; Clarivate Analytics, Boston, MA, USA) to store the results from searches and eliminate duplicates between bibliographic databases. Two researchers screened titles and abstracts independently and discarded studies that were not eligible. We obtained a full text copy of the references considered relevant at this stage to determine their final inclusion. We handled disagreements by discussion between the two researchers and sought arbitration from a third researcher.

Two independent researchers extracted data using a predesigned data extraction form. We collected data to describe the main characteristics of each of the included studies in terms of design, risk of bias, participants, interventions and outcomes assessed. Where more than one publication of one study existed, we grouped the reports together and used the publication(s) with the most complete data according our outcomes of interest. The two researchers reviewed and discussed any disagreements arisen in the data extraction process and, when needed, we sought arbitration from a third researcher.

### Assessment of risk bias

Two researchers assessed the risk of bias of each included study. For clinical trials we used the Cochrane Risk of Bias Assessment Tool and made explicit judgments on selection, performance, detection, attrition and reporting biases [23].

For the rest of study designs we used a modified Risk Of Bias In Non-randomized Studies—of Interventions (ROBINS-I) tool, which is specifically designed to assess non-randomized studies that measure the impact of interventions [31]. We focused the assessment in confounding, selection bias, bias in measurement of interventions, bias due to deviations from intended interventions, bias due to missing data, bias in outcome assessment, and bias in the selection of the reported results.

### Data analysis and synthesis

We obtained effects estimates for our outcomes of interest to ascertain the impact of interim OAT and the magnitude of effect and compare them between studies and the comparisons set at the included studies.

Due to the heterogeneity in outcome measurements, we were not able to pool data with meta-analytical techniques; therefore, we performed a narrative synthesis of the results. We describe the intended analytical plan in the protocol of this review (available at Open Science Framework: https://osf.io/fsvte/).

We rated the quality of evidence for the primary outcomes according GRADE guidance [32] as an expression of the extent to which one can be confident in the effect estimates. We assessed the following domains: risk of bias, directness of evidence, heterogeneity, precision, and publication bias. Each outcome was classified as high, moderate, low or very low depending on the presence of limitations in the mentioned domains. Evidence from randomized trials was considered as high and downgraded on the basis of these limitations. Finally, outcomes with findings from estimates from observational studies were rated as low due to the impact of risk of bias.

## Results

### Search results

**Fig. 1:**
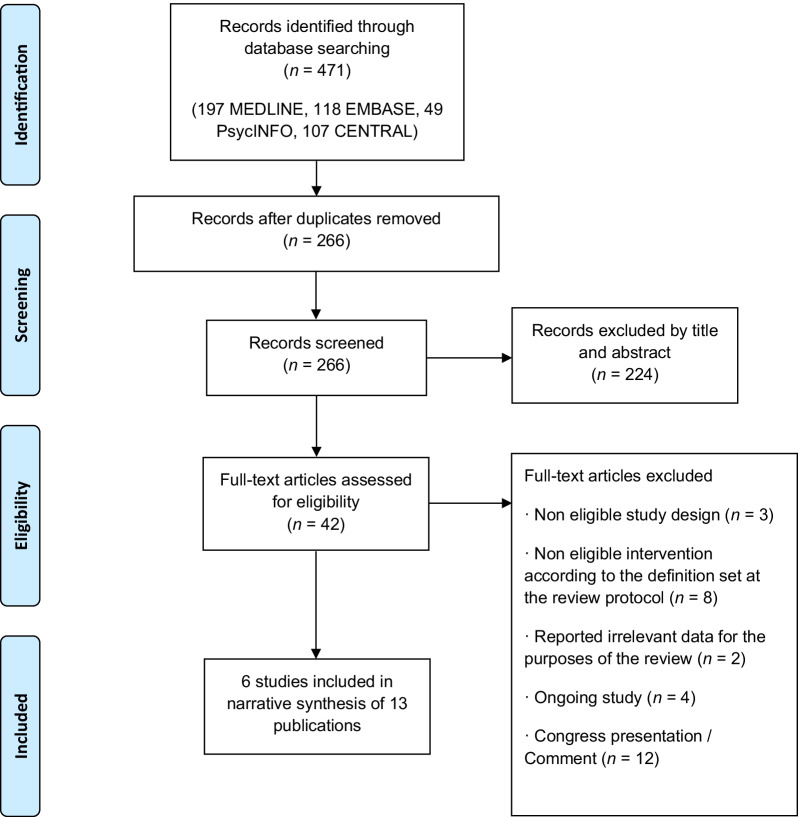
PRISMA 2009 flow diagram of the search and selection process

We identified 471 citations and, after removing duplicates, 266 records were left for screening title and abstract. Once the screening process was completed, we obtained full text copies of 42 articles to decide their final eligibility (Fig. 1).

We excluded 29 studies for the following reasons: 3 publications were excluded due to a non-eligible study design, 8 were excluded because their intervention was not eligible according to the definition of interim OAT, 2 were excluded because they reported irrelevant data for the purposes of this review, 4 were excluded because they are ongoing studies and 12 were excluded because they were conference communications or comments on other articles (Additional file 3).

Finally we included 6 studies that reported their results in 13 publications [21, 33–44] (Additional file 4).

### Characteristics of included studies

We included 5 randomized trials [21, 35, 37, 41, 44] and 1 observational retrospective study [33]. In addition, one of the trials included a publication with an economic evaluation [36]. Table 1 outlines the main characteristics of the included studies that are described in detail at the Additional file 4. All the studies included in this review were carried out in the United States of America, except one that was carried out in Norway [35]. Sample sizes ranged from 50 [41] to 977 participants [33].

**Table 1:** Summary of characteristics of included studies and Risk of Bias (RoB)

Study ID (Design, Country)	Participants	Intervention	Study’s primary outcomes	Risk of Bias (RoB)
Yancovitz et al. 1991 (Randomized Controlled Trial (RCT), USA) [44]	301 participants: 149 Treatment group 152 Frequent contact group	**Interim Clinic**: Methadone administered by a nurse 5 days a week. Saturday medication and one take-home were provided at another site in the same building + AIDS education + free condoms. Minimal counseling on an ad hoc basis. Biweekly urine samples. Maintenance dose of 80 mg/d approximately **Control**: Waiting list, biweekly follow-up and urine samples, and free condoms. Time spent limited to one month and then switched into experimental group	Change in heroin use	Random sequence generation: **Unclear** Allocation concealment: **Low** Blinding of participants and personnel: **High** Blinding of outcome assessment (objective outcomes): **Unclear** Blinding of outcome assessment (subjective outcomes): **Unclear** Incomplete data outcome: **High** Selective reporting bias: **Unclear**
Schwartz et al. 2006 (RCT, USA) [21]	319 participants: 199 Treatment group 120 Waiting list	**Interim Treatment**: Methadone provided under direct observation, participants attended 7 days a week (as the federal regulation required). 3 consecutive unexcused missed doses resulted in discharge from treatment. Mean dose of methadone was 78.4 mg/d **Control**: Waiting list; no further contact with clinical staff unless their name came up on the waiting list	Entry into comprehensive methadone treatment Self-reported days of heroin use Self-reported days of cocaine use Self-reported criminal behavior Number of urine drug test results positive for heroin and cocaine Retention in treatment	Random sequence generation: **Low** Allocation concealment: **Low** Blinding of participants and personnel: **High** Blinding of outcome assessment (objective outcomes): **Low** Blinding of outcome assessment (subjective outcomes): **High** Incomplete data outcome: **High** Selective reporting bias: **Unclear**
NCT02360007 (RCT, USA) [41]	50 participants: 25 Treatment group 25 Waiting list	**Interim Treatment**: Interim buprenorphine treatment (IBT) participants will complete buprenorphine (BUP) induction in Week 1 (or longer if required), during which they will attend the clinic daily. Thereafter, during Weeks 2–12 IBT participants will visit the clinic every two weeks to take their BUP dose, provide a urine specimen and receive their remaining doses in a secure dispenser that makes each day’s dose available during a 3-h window **Control**: Waitlist control (WLC) participants will remain on the waitlist for their treatment of choice. They will visit the clinic to complete follow-up assessments and provide staff-observed urines according to the same schedule as IBT participants Participants in both conditions will complete follow-up assessments and provide a urine specimen at 4, 8, 12, 18 and 24 weeks after trial entry. WLC participants who have not entered treatment by Week 12 will be offered IBT at that time, providing an additional within-subject evaluation of IBT effects. Thus the overall possible study duration may vary between 12—28 weeks	Negative results for illicit opioids	Random sequence generation: **Low** Allocation concealment: **Unclear** Blinding of participants and personnel: **High** Blinding of outcome assessment (objective outcomes): **Unclear** Blinding of outcome assessment (subjective outcomes): **Unclear** Incomplete data outcome: **High** Selective reporting bias: **High**
NCT00712036 (RCT, USA) [37]	230 participants: 99 Treatment group (IM) 104 Control condition 1 (SM) 27 Control condition 2 (RM)	**Interim Treatment (IM)**: Methadone treatment for up to 4 months with emergency counseling only. Treatment was provided following federal regulations. Mean methadone dose of 91.8 mg/d (SD 21.3) **Control**: **1. Scheduled counseling/Standard Methadone Treatment (SM)**: Methadone treatment with counseling as usual. Methadone treatment programs (MTPs) usual practices, which permitted take-home doses contingent upon tenure and progress in treatment (e.g., negative drug tests), required regularly scheduled counseling, treatment planning, other psychosocial treatment as needed, and more frequent drug testing than in IM. Mean methadone dose of 79.4 mg/d (SD 22.3) **2. Restored Methadone Treatment (RM)**: Methadone treatment with counseling provided by a clinician with a lower caseload than counseling as usual. That counselor carried a reduced case load not to exceed 25 patients and was instructed to see the participants as frequently as the participants wanted and the counselor deemed appropriate. Mean methadone dose of 64.7 mg/d (SD 15.6)	Opiate positive drug test	Random sequence generation: **Low** Allocation concealment: **Low** Blinding of participants and personnel: **High** Blinding of outcome assessment (objective outcomes): **Low** Blinding of outcome assessment (subjective outcomes): **High** Incomplete data outcome: **Low** Selective reporting bias: **Low**
Krook et al. 2002 (RCT, Norway) [35]	106 participants: 55 Treatment group 51 Control condition	**Buprenorphine**: Buprenorphine without additional rehabilitation or support. 4 mg Subutex® on the first day, increased to 16 mg/d on the following 8 days. At the 12^th^ week, dosage was scaled down to 4 mg/d **Control**: Placebo without additional rehabilitation or support. 4 mg of Subutex® on the first day and then the dose was decreased to 0 in 9 days and thereafter replaced by placebo	Retention (is the patient still in the project?) Compliance (how many of the total number of doses had been taken?)	Random sequence generation: **Low** Allocation concealment: **Low** Blinding of participants and personnel: **Low** Blinding of outcome assessment (objective outcomes): **Low** Blinding of outcome assessment (subjective outcomes): **Low** Incomplete data outcome: **Unclear** Selective reporting bias: **Low**
Friedmann et al. 1994 (Cohort study, USA) [33]	977 participants: 314 Treatment group 663 Control condition	**Interim Methadone Treatment**: A physical examination on admission was performed by a physician, methadone was administered by a registered nurse six days per week with one take-home dose for Sunday, and education about AIDS-risk reduction was provided. Only crisis counseling services were provided, and the focus of the interim clinic counselors was otherwise limited to AIDS-risk reduction. The clinic had a capacity of 150 patients, thus providing a ratio of 1 counselor to 75 patients **Control**: The comparison group includes all consecutive 1990 and 1991 admissions to Beth Israel's traditional methadone clinics located in the same immediate area as the interim clinic	Retention rate in treatment	Confounding: **Serious** Selection of participants: **Serious** Classification of intervention: **Low** Deviation from intended intervention: **Serious** Missing outcome data: **Low** Measurement of the outcome: **Low** Selection of the reported result: **Low**

All participants in the included trials were adults with an opioid use disorder diagnosed according to DSM (IV or DSM V) and/or were on a waiting list for standard OAT. The observational study [33] included all consecutive admissions to an interim clinic during 2 years.

The interventions assessed in the included studies met the pre-defined definition of interim OAT set in the review protocol (Table 1). Interventions had slight differences among studies, such as the medication used. While most of the studies used methadone, two used buprenorphine [35, 41].

Most of the studies did not allow take-home medication [21, 35, 37] or allowed just one take-home dose per week [33, 44]; nevertheless, one study used a portable dispenser device holding multiple-day doses in individually locked compartments and allowed access to the medication during a 3-h window every day [41].

What made the studies differentiate the most were the control conditions since some included a waiting list for standard OAT as a control condition [21, 41, 44] and others used standard OAT as a control condition [33, 37]. Finally, one of the studies [35] compared two modalities of interim treatment, one with buprenorphine and the other with placebo.

Primary outcomes among the studies mainly included retention in treatment, access to standard OAT, use of illicit drugs and criminal activities or illegal income. Of these, only two outcomes (retention in treatment and access to standard OAT) were a priori considered as main outcomes for the purpose of this review. Outcome measurements across studies were very heterogeneous, and did not allow us to obtain pooled estimates for them.

### Risk of bias

Although most of the included trials had a low risk in selection bias (only one trial [41] did not provide details on the efforts to conceal the participants assignment to study groups), we had some concerns regarding the rest of domains (Table 1). As we had already anticipated in our protocol due to the nature of the intervention, performance bias was at higher risk for all the included trials with the exception of one of them [35]. Regarding detection bias, we distinguished between the assessment of objective and of subjective outcomes since we considered that objective outcomes were less likely to be biased despite the outcome assessors were not blinded. Hence this domain was mainly rated as low risk of bias for objective outcomes and as unclear or high risk for subjective outcomes. We rated attrition bias as high for most of the studies mainly because either some trial did not provide a sample size estimate or they did not reach it, or the reason for the losses was not clear enough. Finally, we rated reporting bias as unknown for most trials due to lack of information. For the observational study, we considered that the risk of bias was serious due to its selection and performance biases.

### Effect of intervention

Due to the huge variability between study characteristics (mostly because their planned interventions and controls and the outcomes measures) we did not perform a pooled analysis from the study results. We provide a narrative summary of the review findings according to its outcomes of interest and the comparisons assessed in the included trials. We present the detailed review findings in Table 2. We also report the quality of evidence for each primary outcome and each comparison of interest in Table 2.

**Table 2:** Findings by outcome

OUTCOME	FINDINGS
*PRIMARY OUTCOMES*	
**Retention in (Interim/Standard) Treatment** (4 studies)	**Interim versus Waiting List for Standard OAT** (1 study) **Moderate** quality of evidence due to risk of bias (the trial did not describe the efforts to conceal the allocation sequence, and had a high performance and attrition risk of bias) **Retention at 3 months** 23/25 versus 20/25 (no significant difference) (NCT02360007) [41] **Interim versus Standard OAT** (2 studies) **Moderate** quality of evidence due to risk of bias (effects estimates at least from one observational study) **Retention at 3 months** 245/314 versus 557/663 (no significant difference) (Friedmann et al. 1994) [33] **Retention at 4 months** 91/99 versus 84/104 with SM (Standard Methadone Treatment) versus 24/27 with RM (Restored Methadone Treatment) (*p* = 0.06) (NCT00712036) [37] **Retention at 6 months** 217/314 versus 504/663 (no significant difference) (Friedmann et al. 1994) [33] **Retention at 12 months** 60/99 versus 57/104 with SM versus 10/27 with RM (*p* = 0.09) (NCT00712036) [37] 173/314 versus 404/663 (no significant difference) (Friedmann et al. 1994) [33] **Interim with Buprenorphine versus Interim with Placebo** (1 study) **Low** quality of evidence due to indirectness (comparison of two interim treatment modalities) and imprecision in effect estimates **Retention at 3 months** 16/55 versus 1/51 (*p* < 0.001) (Krook et al. 2002) [35]
**Access to Standard OAT** (2 studies)	**Interim versus Waiting List for Standard OAT** (2 studies) **Moderate** quality of evidence due to risk of bias (although trials were at low risk of selection bias, were limited in terms of performance, detection and attrition bias) **Access at 4 months** 151/199 versus 25/120 (*p* < 0.001) (Schwartz et al. 2006) [21] **Access at 10 months** 129/199 versus 33/120 (*p* < 0.001) (Schwartz et al. 2006) [21] **Access at 16 months** 107/149 versus 85/152 (*p* < 0.005) (Yancovitz et al. 1991) [44] There were no studies that assessed this outcome for the following comparisons: Interim versus standard OAT Interim with buprenorphine versus interim with placebo
**Quality of Life or Well-Being** (1 study)	**Interim with Buprenorphine versus Interim with Placebo** (1 study) **Low** quality of evidence due to indirectness (comparison of two interim treatment modalities) and imprecision in effect estimates **Subjective well-being at 3 months** [Visual analogue Scale (VAS); 10 = very bad, 0 = very well] 4.82 (SD not reported) versus 5.92 (SD not reported) (*p* < 0.001) (Krook et al. 2002) [35] **Change in well-being at 3 months** − 2.00 (Confidence Interval (CI) − 2.95; − 1.04) versus − 0.43 (CI − 1.32;0.45) (*p* < 0.001) (Krook et al. 2002) [35] **Temporal satisfaction with life scale (TSLS) at 3 months** [TSLS; 15 items, 7 point Likert response, 0 = “very well” and 7 = “very bad”] 4.81 (SD not reported) versus 5.11(SD not reported) (*p* < 0.05) (Krook et al. 2002) [35] **Change in temporal satisfaction with life scale at 3 months** − 0.65 (CI − 1.00; − 0.31) versus − 0.24 (CI − 0.57;0.09) (*p* < 0.05) (Krook et al. 2002) [35] There were no studies that assessed this outcome for the following comparisons: Interim versus waiting list to standard OAT Interim versus standard OAT
**Satisfaction with Treatment** (1 study)	**Interim versus Waiting List for Standard OAT** (1 study) **Very low** quality of evidence due to risk of bias (some concerns about selection bias, and high risk for performance and attrition bias), indirectness (effect estimate obtained only from the intervention arm from one trial) and imprecision in effect estimates **User satisfaction for patients at the interim intervention group at 3 months** [according to a 5-point Likert score, with a higher score corresponding to greater satisfaction] 4.6 (SD 0.7) (NCT02360007) [41] There were no studies that assessed this outcome for the following comparisons: Interim versus standard OAT Interim with buprenorphine versus interim with placebo
*SECONDARY OUTCOMES*	
**Use of Illicit Drugs and/or Non-Prescribed Psychoactive Substances (Heroin Positive Urine Tests)** (4 studies)	**Interim versus Waiting List for Standard OAT** (3 studies) **At 1 month** 22/75 versus 56/94 (Odds Ratio (OR) 3.55; CI 95% 1.862–6.771) (*p* < 0.001) (Yancovitz et al. 1991) [44] **At 3 months** 17/25 versus 0/25 (number of negative tests) (*p* < 0.001) (NCT02360007) [41] **At 4 months** 99/175 versus 80/113 (*p* < 0.001) (Schwartz et al. 2006) [21] **At 10 months** 75/156 versus 73/101 (*p* = 0.001) (Schwartz et al. 2006) [21] **Interim versus Standard OAT** (1 study) **At 4 months** 44/96 versus 47/92 with SM versus 10/25 with RM (*p* = 0.98) (NCT00712036) [37] **At 12 months** 0.46 (Standard error (SE) 0.05) versus 0.48 (SE 0.05) with SM versus 0.51 (SE 0.11) with RM (*p* = 0.91) (NCT00712036) [37] There were no studies that assessed this outcome for the following comparison: Interim with buprenorphine versus interim with placebo
**Use of Illicit Drugs and/or Non-Prescribed Psychoactive Substances (Heroin Self-Reported)** (4 studies)	**Interim versus Waiting List for Standard OAT** (2 studies) **At 1 month** 21/75 versus 83/94 (*p* < 0.001) (Yancovitz et al. 1991) [44] **At 4 months** Days of heroin use in the past 30 days: 4.2 (SD 8.6) versus 26.4 (SD 8.8) (*p* < 0.001) (Schwartz et al. 2006) [21] **At 10 months** Days of heroin use in the past 30 days: 5.7 (SE 0.90) versus 17.7 (SE 1.2) (*p* < 0.001) (Schwartz et al. 2006) [21] **Interim versus Standard OAT** (1 study) **At 4 months** Days of heroin use in the past 30 days: 2.6 (SE 0.5) versus 3.6 (SE 0.8) with SM versus 2.8 (SE 1.0) with RM (*p* = 0.21) (NCT00712036) [37] **At 12 months** Days of heroin use in the past 30 days: 4.4 (SE 0.98) versus 6.2 (SE 1.2) with SM versus 6.9 (SE 2.4) with RM (*p* = 0.57) (NCT00712036) [37] **Interim with Buprenorphine versus Interim with Placebo** (1 study) **At 3 months** 3.99 (SD not reported) versus 6.63 (SD not reported) (using a VAS from 0 = “drug free” to 10 = “daily heavy drug abuse”) (*p* < 0.001) (Krook et al. 2002) [35] Change in self-reported heroin use − 3.21 (CI − 4.29; − 2.13) versus 0.52 (− 0.64; 1.68) (*p* < 0.001) (Krook et al. 2002) [35]
**Use of Illicit Drugs and/or Non-Prescribed Psychoactive Substances** **(Cocaine Positive Urine Tests)** (3 studies)	**Interim versus Waiting List for Standard OAT** (2 studies) **At 1 month** 51/75 versus 66/94 (OR 1.109; CI 95% 0.575–2.138) (*p* = 0.76) (Yancovitz et al. 1991) [44] **At 4 months** 107/174 versus 62/99 At 10 months (*p* = 0.85) (Schwartz et al. 2006) [21] **At 10 months** 79/153 versus 60/101 (no significant differences) (Schwartz et al. 2006) [21] **Interim versus Standard OAT** (1 study) **At 4 months** 32/96 versus 41/92 with SM versus 6/25 with RM (*p* = 0.75) (NCT00712036) [37] **At 12 months** 0.39 (SE 0.05) versus 0.36 (SE 0.05) with SM versus 0.32 (SE 0.10) with RM (*p* = 0.23) (NCT00712036) [37] There were no studies that assessed this outcome for the following comparison: Interim with buprenorphine versus interim with placebo
**Use of Illicit Drugs and/or Non-Prescribed Psychoactive Substances** **(Cocaine Self-Reported)** (3 studies)	**Interim versus Waiting List for Standard OAT** (2 studies) **At 1 month** 29/75 versus 79/94 (*p* < 0.001) (Yancovitz et al. 1991) [44] **At 4 months** Days of cocaine use in the last 30 days: 2.4 (SD 5.5) versus 5.8 (SD 8.8) (*p* < 0.001) (Schwartz et al. 2006) [21] **At 10 months** Days of cocaine use in the last 30 days: 3.5 (SE 0.63) versus 5.8 (SE 0.83) (*p* = 0.001) (Schwartz et al. 2006) [21] **Interim versus standard OAT** (1 study) **At 4 months** Days of cocaine use in the last 30 days: 1.6 (SE 3.8) versus 3.0 (SE 7.3) with SM versus 1.4 (SE 0.8) with RM (*p* = 0.082) (NCT00712036) [37] **At 12 months** Days of cocaine use in the last 30 days: 1.8 (SE 0.62) versus 2.9 (SE 0.74) with SM versus 1.0 (SE 0.82) with RM (*p* = 0.42) (NCT00712036) [37] There were no studies that assessed this outcome for the following comparison: Interim with buprenorphine versus interim with placebo
**Use of Illicit Drugs and/or Non-Prescribed Psychoactive Substances** **(Non-Prescribed Methadone Self-Reported)** (1 study)	**Interim versus Waiting List for Standard OAT** (1 study) **At 1 month** 1/75 versus 37/94 (*p* < 0.001) (Yancovitz et al. 1991) [44] There were no studies that assessed this outcome for the following comparisons: Interim versus standard OAT Interim with buprenorphine versus interim with placebo
**Use of Illicit Drugs and/or Non-Prescribed Psychoactive Substances** **(Other Drugs Self-Reported)** (1 study)	**Interim with Buprenorphine versus Interim with Placebo** (1 study) **At 3 months** 3.56 (SD not reported) versus 4.4 (SD not reported) (using a VAS from 0 = “drug free” to 10 = “daily heavy drug abuse”) (*p* < 0.01) (Krook et al. 2002) [35] Change in self-reported use of other drugs 0.66 (CI − 1.77; 0.44) versus 1.11 (CI 0.18; 2.05) (*p* < 0.01) (Krook et al. 2002) [35] There were no studies that assessed this outcome for the following comparisons: Interim versus waiting list to standard OAT Interim versus standard OAT
**Criminal Activities/Illegal Income** **(Illegal Income)** (2 studies)	**Interim versus Waiting List for Standard OAT** (1 study) **At 4 months** 36$ (SD 160) versus 412$ (SD 1391) (*p* < 0.02) (Schwartz et al. 2006) [21] **At 10 months** 40$ (SE 18.21) versus 135$ (SE 23.69) (*p* = 0.018) (Schwartz et al. 2006) [21] **Interim versus Standard OAT** (1 study) **At 4 months** 8$ (SE 3) versus 336$ (SE 287) with SM versus 113$ (SE 113) with RM (*p* < 0.001) (NCT00712036) [37] **At 12 months** 27$ (SE 12) versus 55$ (SE 19) with SM versus 14$ (SE 14) with RM (*p* < 0.001) (NCT00712036) [37] There were no studies that assessed this outcome for the following comparison: Interim with buprenorphine versus interim with placebo
**Criminal Activities/Illegal Income** **(Illegal Activities)** (2 studies)	**Interim versus Waiting List for Standard OAT** (1 study) **At 4 months** 1.7 days (SE 0.60) versus 6.9 (SE 0.79) (*p* < 0.001) (Schwartz et al. 2006) [21] **At 10 months** 2.1 days (SE 0.67) versus 7.3 (SE 0.88) (p < 0.001) (Schwartz et al. 2006) [21] **Interim versus Standard OAT** (1 study) **At 4 months** 0.48 days (SE 0.29) versus 1.11 (SE 0.47) with SM versus 1.13 (SE 1.13) with RM (*p* = 0.003) (NCT00712036) [37] **At 12 months** 0.96 days (SE 0.47) versus 2.00 (SE 0.66) with SM versus 1.30 (SE 1.20) with RM (*p* = 0.46) (NCT00712036) [37] There were no studies that assessed this outcome for the following comparison: Interim with buprenorphine versus interim with placebo
**Criminal Activities/Illegal Income** **(Arrests)** (1 study)	**Interim versus Waiting List for Standard OAT** (1 study) **At 6 months** Number of participants arrested: 31/198 versus 24/119 (*p* = 0.18) (Schwartz et al. 2006) [21] Mean number of arrests: 0.2 arrests (SE 0.06) versus 0.34 arrests (SE 0.09) (*p* = 0.02) (Schwartz et al. 2006) [21] **At 12 months** Number of participants arrested: 53/198 versus 31/119 (*p* = 0.96) (Schwartz et al. 2006) [21] Mean number of arrests: 0.33 (SE 0.09) versus 0.39 (SE 0.11) (*p* = 0.16) (Schwartz et al. 2006) [21] **At 24 months** Number of participants arrested: 77/198 versus 54/119 (*p* = 0.75) (Schwartz et al. 2006) [21] Mean number of arrests: 0.61 (SE 0.14) versus 0.76 (SE 0.18) (*p* = 0.16) (Schwartz et al. 2006) [21] There were no studies that assessed this outcome for the following comparisons: Interim versus standard OAT Interim with buprenorphine versus interim with placebo
**Criminal Activities/Illegal Income** **(Severe Crimes)** (1 study)	**Interim versus Waiting List for Standard OAT** (1 study) **At 6 months** 6/198 versus 1/119 (*p* = 0.23) (Schwartz et al. 2006) [21] **At 12 months** 7/198 versus 3/119 (*p* = 0.62) (Schwartz et al. 2006) [21] **At 24 months** 10/198 versus 6/119 (*p* = 1.0) (Schwartz et al. 2006) [21] There were no studies that assessed this outcome for the following comparisons: Interim versus standard OAT Interim with buprenorphine versus interim with placebo
**Mental/Physical Health** **(Mental Health)** (3 studies)	**Interim versus Waiting List for Standard OAT** (1 study) **Global Severity Index (GSI) above cut-off at 3 months** [a widely used indicator for distress, using a cut-off > or = 63] 7/23 versus 13/25 (significance not reported) (NCT02360007) [41]; this trial also reports more data on mental health (Beck Anxiety Inventory, Beck Depression Inventory, Brief Symptom Inventory and Addiction Severity Index (ASI) Psychiatric composite score) but further data could not be extracted because the authors reported the significances of the change in mean measures, but not the actual measures **Interim versus Standard OAT** (1 study) **Psychiatric ASI composite score at 4 months** [score ranging from 0 = “no problem” to 1 = “extreme problem”] 0.05 (SE 0.01) versus 0.02 (SE 0.01) with SM versus 0.01 (SE 0.02) with RM (*p* = 0.75) (NCT00712036) [37] **Psychiatric ASI composite score at 12 months** 0.06 (SE 0.02) versus 0.06 (SE 0.02) with SM versus 0.02 (SE 0.03) with RM (*p* = 0.61) (NCT00712036) [37] **Interim with Buprenophine versus Interim with Placebo** (1 study) **Anxiety and depression at 3 months** [measured with Symptom Checklist-5 on a four-point scale ranging from 1 = ‘not at all’ to 4 = ‘extremely’] 2.51 (SD not reported) versus 2.63 (SD not reported) (no significant difference) (Krook et al. 2002) [35] **Change in anxiety and depression (at 3 months)** − 0.3 (CI − 0.52; − 0.08) versus − 0.17 (CI − 0.40; 0.07) (no significant difference) (Krook et al. 2002) [35]
**Mental/Physical Health** **(Physical Health)** (2 studies)	**Interim versus Waiting List for Standard OAT** (1 study) One study (Schwartz et al. 2006) [21] reports on Human Immunodeficiency Virus (HIV) risk behaviors, but data could not be extracted as the authors only report *p*-values and statistic test results **Interim versus Standard OAT** (1 study) **Medical ASI Composite score at 4 months** [score ranging from 0 = “no problem” to 1 = “extreme problem”] 0.13 (SE 0.03) versus 0.10 (SE 0.03) with SM versus 0.19 (SE 0.06) with RM (*p* = 0.70) (NCT00712036) [37] **HIV risk in injector subsample at 4 months** 0.08 (SE 0.06) times injected with unsterilized needles versus 0.00 (SE 0.05) with SM versus 0.04 (SE 0.04) with RM (*p* > 0.05) (NCT00712036) [37] **Medical ASI Composite score at 12 months** 0.19 (SE 0.03) versus 0.12 (SE 0.03) with SM versus 0.12 (SE 0.06) with RM (*p* = 0.56) (NCT00712036) [37] **HIV risk in injector subsample at 12 months** 0.00 (SE 0.00) times injected with unsterilized needles versus 0.00 (SE 0.0) with SM versus 0.00 (SE 0.0) with RM (*p* > 0.05) (NCT00712036) [37] There were no studies that assessed this outcome for the following comparison: Interim with buprenorphine versus interim with placebo
**Adverse Effects** (2 studies)	**Interim versus Standard OAT** (1 study) **Number of participants with at least one serious adverse effect** at **12 months** 19/99 versus 9/104 with SM versus 4/27 with RM (NCT00712036) [37] **Interim with Buprenophine versus Interim with Placebo** (1 study) One trial narratively reported that no deaths or other serious side effects were observed during the 3 months of follow-up but provided no data (Krook et al. 2002) [35] There were no studies that assessed this outcome for the following comparison: Interim versus waiting list to standard maintenance treatment

#### Retention in interim OAT

Three trials [35, 38, 41] and one observational study [33] reported on retention at or before 4 months of treatment. This can be interpreted as a measure of retention in the interim OAT itself since this treatment approach lasts a maximum of 120 days.

One trial did not find significant differences in retention rates between interim OAT and a group of patients in a waiting list for standard OAT (92% vs. 80%) [41]. Two studies [33, 38] compared interim OAT with standard OAT. One trial [38] found no significant differences in treatment retention rates at 4 months between the 3 comparison groups (91.9%, 80.8%, and 88.9% respectively for interim methadone treatment (IM), standard methadone treatment (SM) and restored methadone treatment (RM); see Table 1 and Additional file 4 for a detailed description). Similarly, the observational study [33] did not find significant differences with regards to retention rate at 3 months (78% vs. 84%). For the comparisons of interim OAT versus waiting lists or standard OAT we rated the quality of evidence as moderate for the impact of risk of bias.

Finally, one study [35] comparing interim OAT with buprenorphine to interim treatment with placebo found significant differences in the proportion of patients remaining in treatment at 12 weeks (16 vs. 1 patients, *p* < 0.001) and in the mean number of days of participation in treatment (42 vs. 14 days), with the intervention group showing higher retention rates in both cases. We rated the quality of evidence as low for this comparison due to indirectness (the trial compared two interim treatment modalities) and the imprecision of effect estimates.

#### Access to standard OAT

Two trials comparing interim OAT with a waiting list for standard OAT [21, 44] evaluated the access to standard OAT. One of these studies [21] found a significant difference when assessing this outcome at both 4 and 10 months after starting the treatment. At 4 months, 75.9% of the patients in the intervention group accessed a standard OAT while only 20.8% from the control group did (*p* < 0.001). At 10 months this difference remained significant (64.8% vs. 27.5%; *p* < 0.001). In total, at the end of the study, the authors reported that 78.4% of the participants in interim OAT group had entered standard OAT compared to 32.5% in the comparison group.

The other study [44] also found significant differences (*p* < 0.005) in this outcome measured 16 months after the beginning of the study, with 72% of patients in the intervention group and 56% in the control group accessing standard OAT. We rated the quality of evidence as moderate for this outcome due to the impact of risk of bias.

#### Retention in standard OAT

Two studies comparing interim OAT with standard OAT, one trial [37] and one observational study [33], evaluated the retention in standard OAT at 12 months after the beginning of the study. The trial [37] found no significant differences between groups for this outcome (60.6% for IM, 54.8% for SM, and 37.0% for RM). Similarly, the observational study [33] did not show any significant differences in retention rates at 12 months when comparing interim OAT with standard OAT (55% vs. 61%, *p* = 0.17). We rated the quality of evidence as moderate for this outcome due to the impact of risk of bias.

#### Quality of life or well-being

None of the studies comparing the impact of an interim OAT with a waiting list or standard OAT reported on quality of life. Only the trial that compared interim OAT with buprenorphine to interim treatment with placebo [35] assessed this outcome at 3 months of follow-up with a visual analogic scale and a validated instrument. Both scales showed a greater and significant increase in well-being for the buprenorphine group compared to the placebo group. As we did not obtain results from trials comparing interim OAT versus waiting lists or standard OAT we rated the quality of the evidence as low for this outcome due to the impact of indirectness.

#### Satisfaction with treatment

Only one study comparing interim OAT to a waiting list for standard OAT [41] assessed patient satisfaction with treatment at 3 months of follow-up. The authors used a 5 point scale with a higher score corresponding to greater satisfaction and reported a mean score for participants at interim OAT of 4.6 (SD 0.7) at 3 months. However, this was only measured on the intervention group, as for this reason we rated the quality of evidence for this outcome as very low due to the impact of risk of bias, indirectness and the imprecision of results.

#### Use of illicit drugs and/or non-prescribed psychoactive substances

All the trials reported this outcome but it was the most heterogeneously measured outcome from those of interest. The trials provided information about self-reported use of drugs or urine drug test results for heroin, cocaine, non-prescribed methadone and other drugs.

For heroin use determined by urine drug test, three trials comparing interim OAT with a waiting list for standard OAT found significant group differences either at 1 month [44], at 3 months [41], at 4 months [21] or at 10 months [40] of follow-up. Two of those trials also found significant differences in the self-reported use of heroin at 1 month [44], 4 months and 10 months [21] of follow-up. For both outcomes, the intervention group showed better results. One trial comparing interim OAT with standard OAT [37] assessed both outcomes and found no significant differences in their change over time between groups at either 4 and 12 months of follow-up. Finally, the trial comparing interim OAT with buprenorphine with interim treatment with placebo [35] only assessed self-reported heroin use at 3 months and found a significant difference in the change of this outcome in favor of the intervention group.

Regarding cocaine use measured with urine drug tests, two trials comparing interim OAT with a waiting list for standard OAT found no significant group differences at 1 month [44], 4 months [21] or 10 months [40] of follow-up. Nevertheless the same trials found a significant group difference when cocaine use was self-reported. For those two outcomes, one trial comparing interim OAT with standard OAT [37] also found no significant group differences in their change over time either at 4 or at 12 months.

Only one study comparing interim OAT with a waiting list for standard OAT [44] reported on the use of non-prescribed methadone at one month. The authors found a trend toward increased non-prescribed methadone use in the control group. The full results for this outcome are described in Table 2.

#### Criminal activities and/or illegal income

This outcome was reported in two trials but was also quite heterogeneously measured. One trial comparing interim OAT with a waiting list for standard OAT [21] measured the amount of money the participants obtained from illegal sources and the days spent in illegal activities and reported a significant difference for both outcomes at 4 and 10 months of follow-up, favoring the intervention group. Similarly, another trial comparing interim OAT with standard OAT [37] that measured these two outcomes at 4 and 12 months found a significant difference in their change over time in favor of the intervention group for most measures except for illegal activities at 12 months where no significant differences were found.

The number of arrests and severe crimes at 6, 12 and 24 months was also assessed in one trial comparing interim OAT with a waiting list for standard OAT [39] and found no significant differences between groups for most of these measures.

#### Mental and/or physical health

Three trials reported results on mental health, all of them measuring the outcome with different instruments. One trial comparing interim OAT with a waiting list for standard OAT [42] reported changes in psychiatric symptoms. All measures showed that the mean changes were significantly different between the intervention and control group at 3 months of follow-up, with the intervention group showing an improvement in psychiatric symptoms. Nevertheless, the magnitude of this effect could not be assessed as the trial only reported *p*-values and statistical test results. On the other hand, for the other two comparisons, the trials that assessed this outcome [35, 37] did not find any statistical differences between groups.

Two trials, one comparing interim OAT with a waiting list for standard OAT [43] and the other one comparing interim OAT with standard OAT [34] reported results on physical health. The first trial reported on Human Immunodeficiency Virus (HIV) risk behaviors but data could not be extracted and the authors reported ambiguous conclusions about the effect of this intervention. The latter trial also measured HIV risk behaviors and found no significant differences between groups in frequency of injection with unsterilized needles in the injector subsample at either 4 or 12 months. This trial also reported on this outcome through the Addiction Severity Index (ASI) medical composite score and found no significant differences between groups.

#### Adverse effects

No studies comparing interim OAT with a waiting list for standard OAT reported this outcome. One trial comparing interim OAT with standard OAT [37] reported the number of serious adverse events and the number of participants with at least one serious adverse event at 12 months but the authors considered that none of them were study related and they did not report differences between groups. One trial comparing interim OAT with buprenorphine with interim treatment with placebo narratively reported that no deaths or other serious side effects were observed during the 3 months of follow-up.

#### Costs

One trial comparing interim OAT with standard OAT [36] reported the results of a benefit cost analysis with a societal perspective calculated in 2010 US dollars (USD), estimating health service utilization, criminal related costs, and other factors linked to employment in their trial sample. The authors estimated a cost of providing interim OAT of around 3.5 USD per week or a weighted mean total cost of 2,052 USD. The net benefit resulting from a reduction in days in residential treatment, incarceration, arrests was equivalent to 5,939 USD and the benefit–cost ratio was 3.9. The weighted mean cost for the standard OAT options combined was 3,411 USD, with a net benefit of − 2246 USD and a benefit–cost ratio of 0.3. However, there were no significant differences between treatment conditions in total costs benefits during the 12 months of follow up (mean difference between treatment arms of 2155 USD, 95% CI − 582 to 5015 USD; *p* > 0.05). The major contributors to the net benefit showed for the total study sample after the follow-up were related to the reduction of days of incarceration and increases in legal income, but the researchers also observed an increase in days of hospitalization.

## Discussion

Our systematic review identified 6 studies that evaluated the intervention of interest against three different comparisons in terms of a set of outcomes which have also been measured in very different ways and at different follow-up times. Therefore the available body of evidence to inform on the impact of interim OAT is very heterogeneous and did not allow us to obtain a common effect estimate for the outcomes of interest.

We defined four primary outcomes, which are mainly clinical and patient-centered. Most included studies [33, 35, 37, 41] reported on retention in treatment at 4 months or less (which we treated as retention in interim OAT), but few [33, 37] reported retention in treatment at 12 months (which we treated as retention in standard OAT). Our findings suggest that interim OAT does not differ from standard OAT in terms of retention in treatment. The intervention did not result in higher retention rates at short term (i.e., in interim OAT) compared to a waiting list [41] or the participation in a standard OAT [33, 37]. Only one study offering buprenorphine showed better retention in the interim treatment compared to placebo [35]. The only trial that measured retention in standard OAT did not show differences between the interim and standard OAT [37]. The overall quality of evidence for this outcome is moderate due to some concerns regarding the risk of bias from included trials.

Two trials assessed access to standard OAT and showed significant differences when comparing interim OAT with a waiting list for a standard OAT with moderate quality of evidence thus suggesting that patients in interim OAT would be more likely to access a standard OAT than those who are on a waiting list.

It is worth mentioning that the other two outcomes that we defined as primary outcomes—quality of life and satisfaction with treatment—have not really been investigated. Only one trial assessed quality of life and another one assessed satisfaction with treatment. We chose those outcomes because from a patient-centered perspective it is important to include a more comprehensive view of treatment outcomes [45]. Nevertheless the measurement and standardization of these outcomes has proven to be challenging as there is no consensus on which outcome measures of functioning or quality of life really relate to drug use and is a topic that still generates great debate [46]. Moreover, there is still no consensus in what perspective should be prioritized to better reflect the benefits of treatment (e.g., social negative outcomes to be avoided, symptom reduction, or patient perspective). In fact, there is still an active, ongoing debate in the addiction treatment research field surrounding both the most appropriate consumption outcome measure [47, 48] and whether (and which, if any) non-consumption outcome measures should be incorporated [49, 50].

Our secondary outcomes are mainly related to drug use and social impact although we also assessed other relevant domains beyond drug use, including psychiatric and medical status, safety and costs. Three trials assessed heroin use with urine drug tests at different follow-up times and found that interim OAT reduced its use significantly when compared with a waiting list for standard OAT [21, 41, 44]. On the other hand, another trial did not find differences in this outcome between interim and standard OAT, thus suggesting that initiating OAT with interim OAT may be as effective as initiating it directly with standard OAT in regards of heroin use reduction during the first year of follow-up and better than remaining on a waiting list. There is not as much evidence and the results are not so clear when it comes to social impact. Interim OAT seems to be more effective than a waiting list for standard OAT in reducing the amount of money obtained from illegal sources and the days spent in illegal activities, but showed no differences in the number of arrests and severe crimes. In addition, interim OAT seems to have similar results compared to standard OAT at reducing the amount of money obtained from illegal sources and the days spent in illegal activities, but seems to achieve these results faster. The impact of interim OAT on mental and physical health is not as widely evaluated and results are more ambiguous. Interim OAT might be more effective to improve mental health outcomes than a waiting list for standard OAT (although these results were not assessed for a long term follow-up), but showed no differences compared to interim treatment with placebo. Interim OAT did not cause more adverse events when compared to standard OAT nor with interim treatment with placebo, which reinforces its safety profile. And, finally, there were no statistical significant differences in terms of benefit cost analysis.

Our results align with those from a previous literature review [51] that summarized some articles evaluating interim OAT and found that interim OAT is better than a waiting list for a standard OAT and no worse than standard OAT in terms of retention, heroin use and criminal activities among others. Nevertheless, our review also reports on other relevant outcomes, was conducted systematically, assessed the risk of bias for each included study, and goes beyond a description of the included studies by synthesizing the results together. A couple of Cochrane reviews on OAT [10, 11] also included some of the trials in this review and found similar results in terms of treatment retention, heroin use or criminal activities [21, 35, 44]. Notwithstanding, these reviews included different approaches of OAT together and we have specifically focused on assessing the impact of interim OAT.

## Strengths and limitations

We conducted a review according pre-specified criteria and standardized methods that were publicly registered and identified studies through a comprehensive search to assess the impact of the intervention of interest in terms of patient centered outcomes. In results of that, our findings provide reliable data to inform policy makers and other stakeholders in their decisions.

Nevertheless we acknowledge some limitations. Due to the heterogeneity in comparisons and outcome measurements of the primary studies we have not been able to perform a quantitative analysis and therefore our conclusions do not provide definitive estimates of effect.

## Implications for practice and research

The results of our review are probably more directed to policy-makers working in public health than practitioners. With the current evidence, policy-makers operating in contexts where there is insufficient coverage and long waiting lists for standard OAT and/or high rates of opioid use disorder should include interim OAT in their health policies.

Furthermore, with this evidence, it seems reasonable to say that lowering treatment thresholds for a period of time, as some other authors have previously suggested in the broader area of substance abuse treatment [52], is not only not detrimental for patients but can also be beneficial when the only other options are a waiting list or no treatment at all. Amidst the current global opioid crisis, this is of particular relevance for contexts with a markedly high prevalence of opioid use and/or low availability of standard OAT due to a diversity of reasons such as insufficient service providers, strict regulations or insufficient funds. In a situation of shortage of standard OAT, where treatment demand exceeds availability, interim OAT may be an essential asset to avoid unnecessary delays once the request for OAT is made and the indication is established. Other mechanisms and processes—within and outside current regulatory systems—which could expand and facilitate access and entry of people with opioid use disorder into OAT are also worth considering and studying [53, 54].

In any case, further research is needed to establish a minimum, common set of outcomes to be assessed in interim OAT research, and to identify the subgroup of people with opioid use disorder most likely to benefit from such treatment.

## Conclusions

Interim OAT likely results more effective than a waiting list for standard OAT in regards of access to standard OAT, reduction in heroin use and criminal activities, and improvement in mental health. Nevertheless the evidence is uncertain about the effect of interim OAT on physical health. Moreover, interim OAT probably is as effective as standard OAT in regards to retention in treatment (both short and long term), reduction in heroin and cocaine use, reduction in criminal activities and improvement of mental and physical health without increasing adverse events. Interim OAT with buprenorphine is also likely more effective than interim treatment with placebo in regards of short term treatment retention and reduction in heroin use and has no differences in regards to adverse events. The effect of interim OAT on quality of life and satisfaction with treatment is very uncertain as it has not been much investigated.

## Supplementary Information

**Additional file 1.** PRISMA 2009 checklist.

**Additional file 2.** Search strategy.

**Additional file 3.** Reasons for exclusion of studies.

**Additional file 4.** Table of characteristics of included studies.

## Data Availability

All data generated or analyzed during this study are presented in the primary research articles reviewed or in this published article and its supplementary information files.

## Abbreviations

AIDS: Acquired immune deficiency syndrome
ASI: Addiction Severity Index
CENTRAL: Cochrane Central Register of Controlled Trials
DSM: Diagnostic and Statistical Manual of mental disorders
EMBASE: Excerpta Medica dataBASE
FDA: Food and Drug Administration
GRADE: Grading of Recommendations Assessment, Development, and Evaluation
HIV: Human immunodeficiency virus
ICD: International Classification of Diseases
IM: Interim methadone treatment
MEDLINE: MEDical LIterature analysis and retrieval system onliNE
OAT: Opioid agonist treatment
PRISMA: Preferred Reporting Items for Systematic reviews and Meta-Analyses
PROSPERO: International PROSPEctive Register Of systematic reviews
PsycINFO: Psychological INFOrmation database
RM: Restored methadone treatment
ROBINS-I: Risk Of Bias In Non-randomised Studies—of Interventions
SM: Standard methadone treatment
USD: US dollars
