# Supplementary material for: Interim opioid agonist treatment for opioid addiction: a systematic review
Source: Harm Reduct J. 2022 Jan 29;19:7. doi: 10.1186/s12954-022-00592-x (PMC8800211; doi:10.1186/s12954-022-00592-x)
Supplement: Supplementary file 2 — Additional file 2. Search strategy. [file 12954_2022_592_MOESM2_ESM.docx]

# Additional file 2 Search strategy

**MEDLINE**

Ovid MEDLINE(R) and Epub Ahead of Print, In-Process & Other Non-Indexed Citations and Daily <1946 to February 12, 2019>

13/02/2019

1 exp METHADONE/ (11864)

2 exp BUPRENORPHINE/ (4794)

3 exp NALOXONE/ (24453)

4 exp Opiate Substitution Treatment/ (2369)

5 interim.ti,ab. (13986)

6 (1 or 2 or 3 or 4) and 5 (49)

7 (interim adj5 methadone).ti,ab. (22)

8 (interim adj5 buprenorphine).ti,ab. (10)

9 (interim adj9 opioid* adj3 maintenance).ti,ab. (0)

10 (interim adj9 agonist* adj3 maintenance).ti,ab. (0)

11 (interim adj9 opioid* adj3 depend*).ti,ab. (4)

12 (interim adj9 heroin adj3 depend*).ti,ab. (0)

13 (methadone adj20 without adj5 counsel*).ti,ab. (12)

14 (buprenorphine adj20 without adj5 counsel*).ti,ab. (2)

15 (minim* adj7 methadone adj7 service*).ti,ab. (5)

16 (methadone adj1 alone).ti,ab. (69)

17 6 or 7 or 8 or 9 or 10 or 11 or 12 or 13 or 14 or 15 or 16 (133)

**MEDLINE**

PubMed

21/05/2020

#1 "METHADONE"[Mesh] 12,334

#2 "BUPRENORPHINE"[Mesh] 5,269

#3 "NALOXONE"[Mesh] 25,251

#4 "Opiate Substitution Treatment"[Mesh] 2,919

#5 interim[tiab] 15,309

#6 (#1 OR #2 OR #3 OR #4) AND #5 54

#7 interim[tiab] AND methadone[ti] 25

#8 interim[tiab] AND buprenorphine[ti] 12

#9 interim[tiab] AND opioid*[ti] AND maintenance[ti] 1

#10 interim[tiab] AND agonist*[ti] AND maintenance[ti] 0

#11 interim[tiab] AND opioid*[ti] AND dependen*[ti] 4

#12 interim[tiab] AND heroin[ti] AND dependen*[ti] 4

#13 methadone[ti] AND without[ti] AND counsel*[ti] 0

#14 methadone[ti] AND without[tiab] AND counsel*[tiab]0

#15 buprenorphine[ti] AND without[tiab] AND counsel*[tiab] 0

#16 minim*[tiab] AND methadone[ti] AND service*[ti] 7

#17 methadone[ti] AND alone[ti] 17

#18 #7 OR #8 OR #9 OR #10 OR #11 OR #12 OR #13 OR #14 OR #15 OR #16 OR #17 64

**EMBASE**

embase.com

13/03/2020

#1 'methadone'/exp 32,539

#2 'buprenorphine'/exp 17,46

#3 'naloxone'/exp 41,77

#4 'opiate substitution treatment'/exp 2,246

#5 interim:ti,ab 34,263

#6 (#1 OR #2 OR #3 OR #4) AND #5 106

#7 (interim NEAR/5 methadone):ti,ab 32

#8 (interim NEAR/5 buprenorphine):ti,ab 18

#9 (interim NEAR/9 opioid* NEAR/3 maintenance):ti,ab 1

#10 (interim NEAR/9 agonist* NEAR/3 maintenance):ti,ab 0

#11 (interim NEAR/9 opioid* NEAR/3 depend*):ti,ab 4

#12 (interim NEAR/9 heroin NEAR/3 depend*):ti,ab 2

#13 (methadone NEAR/20 without NEAR/5 counsel*):ti,ab 11

#14 (buprenorphine NEAR/20 without NEAR/5 counsel*):ti,ab 5

#15 #6 OR #7 OR #8 OR #9 OR #10 OR #11 OR #12 OR #13 OR #14 118

**PsycINFO**

EBSCOHost

21/05/2019

S1 DE "Methadone" (4,821)

S2 DE "Buprenorphine" (2,204)

S3 DE "Naloxone" (4,639)

S4 DE "Methadone Maintenance" (3,610)

S5 TI interim OR AB interim (2.045)

S6 (TI interim OR AB interim) AND (S1 OR S2 OR S3 OR S4) (37)

S7 TI interim N5 methadone OR AB interim N5 methadone (20)

S8 TI interim N5 buprenorphine OR AB interim N5 buprenorphine (7)

S9 TI interim N9 opioid* N5 maintenance OR AB interim N9 opioid* N5 maintenance (1)

S10 TI interim N9 agonist* N5 maintenance OR AB interim N9 agonist* N5 maintenance (0)

S11 TI interim N9 opioid* N5 depend* OR AB interim N9 opioid* N5 depend* (2)

S12 TI interim N9 heroin N5 depend* OR AB interim N9 heroin N5 depend* (2)

S13 TI methadone N20 without N5 counsel* OR AB methadone N20 without N5 counsel* (11)

S14 TI methadone N2 alone OR AB methadone N2 alone (45)

S15 S6 OR S7 OR S8 OR S9 OR S10 OR S11 OR S12 OR S13 OR S14 (49)

**Cochrane Central Register of Controlled Trials**

Issue 5 of 12, May 2020

The Cochrane Library

21/05/2019

#1 MeSH descriptor: [Methadone] explode all trees 1240

#2 MeSH descriptor: [Buprenorphine] explode all trees 1126

#3 MeSH descriptor: [Naloxone] explode all trees 2394

#4 interim:ti,ab 11474

#5 (#1 OR #2 OR #3) AND #4 38

#6 (interim NEAR/5 methadone):ti,ab 30

#7 (interim NEAR/5 buprenorphine):ti,ab 15

#8 (interim NEAR/9 opioid* NEAR/3 maintenance):ti,ab 2

#9 (interim NEAR/9 agonist* NEAR/3 maintenance):ti,ab 0

#10 (interim NEAR/9 opioid* NEAR/3 depend*):ti,ab 2

#11 (interim NEAR/9 heroin* NEAR/3 depend*):ti,ab 2

#12 (methadone NEAR/20 without NEAR/5 counsel*):ti,ab 13

#13 (buprenorphine NEAR/20 without NEAR/5 counsel*):ti,ab 4

#14 (minim* NEAR/7 methadone NEAR/7 service*) 5

#15 (methadone NEAR/2 alone):ti,ab 39

#16 #5 OR #6 OR #7 OR #8 OR #9 OR #10 OR #11 OR #12 OR #13 OR #14 OR #15 107
