# Supplementary material for: Interim opioid agonist treatment for opioid addiction: a systematic review
Source: Harm Reduct J. 2022 Jan 29;19:7. doi: 10.1186/s12954-022-00592-x (PMC8800211; doi:10.1186/s12954-022-00592-x)
Supplement: Supplementary file 3 — Additional file 3. Reasons for exclusion of studies. [file 12954_2022_592_MOESM3_ESM.docx]

# Additional file 3 Reasons for exclusion of studies

| **Reason for exclusion** | **Reference** |
| --- | --- |
| Non eligible study design | Sigmon SC, C Meyer A, Hruska B, Ochalek T, Rose G, Badger GJ, Brooklyn JR, Heil SH, Higgins ST, Moore BA, Schwartz RP. Bridging waitlist delays with interim buprenorphine treatment: initial feasibility. Addict Behav. 2015 Dec;51:136-42. doi: 0.1016/j.addbeh.2015.07.030. Epub 2015 Jul 29. PubMed PMID: 26256469; PubMed Central PMCID: PMC4558243. |
|  | Schwartz RP, Jaffe JH, O'Grady KE, Das B, Highfield DA, Wilson ME. Scaling-up interim methadone maintenance: treatment for 1,000 heroin-addicted individuals. J Subst Abuse Treat. 2009 Dec;37(4):362-7. doi: 10.1016/j.jsat.2009.04.002. Epub 2009 Jun 21. PubMed PMID: 19540702; PubMed Central PMCID: PMC2796977. |
|  | Peck KR, Ochalek TA, Badger GJ, Sigmon SC. Effects of Interim Buprenorphine Treatment for opioid use disorder among emerging adults. Drug Alcohol Depend. 2020;208:107879. doi:10.1016/j.drugalcdep.2020.107879 |
| Non eligible intervention according to the definition set at the review protocol | Kidorf M, Brooner RK, Leoutsakos JM, Peirce J. Treatment initiation strategies for syringe exchange referrals to methadone maintenance: A randomized clinical trial. Drug Alcohol Depend. 2018 Jun 1;187:343-350. doi: 10.1016/j.drugalcdep.2018.03.009. Epub 2018 Apr 16. PubMed PMID: 29709732. |
|  | Håkansson A, Widinghoff C, Abrahamsson T, Gedeon C. Correlates of Nine-Month Retention following Interim Buprenorphine-Naloxone Treatment in Opioid Dependence: A Pilot Study. J Addict. 2016;2016:6487217. doi: 10.1155/2016/6487217. Epub 2016 Jan 21. PubMed PMID: 26904355; PubMed Central PMCID: PMC4745813. |
|  | Abrahamsson T, Widinghoff C, Lilliebladh A, Gedeon C, Nilvall K, Hakansson A. Interim buprenorphine treatment in opiate dependence: A pilot effectiveness study. Subst Abus. 2016;37(1):104-9. doi: 10.1080/08897077.2015.1065541. Epub 2015 Jul 15. PubMed PMID: 26176490. |
|  | Senay EC, Jaffe JH, Dimenza S, Renault PF. A 48-week study of methadone, methadyl acetate, and minimal services. 1974 [proceedings]. NIDA Res Monogr. 1976 Jul;(8):88-9. PubMed PMID: 792707. |
|  | Senay E, Jaffe J, diMenza S, Renault P. A 48-week study of methadone, methadyl acetate, and minimal services. Opiate Addiction: Origins and Treatment 1974; (8):185â€201. |
|  | Schwartz RP, Kelly SM, Mitchell SG, O'Grady KE, Sharma A, Jaffe JH. Methadone treatment of arrestees: A randomized clinical trial. Drug Alcohol Depend. 2020;206:107680. doi:10.1016/j.drugalcdep.2019.107680 |
|  | Kelly SM, Schwartz RP, O'Grady KE, et al. Impact of methadone treatment initiated in jail on subsequent arrest. J Subst Abuse Treat. 2020;113:108006. doi:10.1016/j.jsat.2020.108006 |
|  | Scheibe A, Shelly S, Gerardy T, et al. Six-month retention and changes in quality of life and substance use from a low-threshold methadone maintenance therapy programme in Durban, South Africa. Addict Sci Clin Pract. 2020;15(1):13. Published 2020 Feb 21. doi:10.1186/s13722-020-00186-7 |
| Reported irrelevant data for the purposes of the review | Kelly SM, Oʼgrady KE, Jaffe JH, Gandhi D, Schwartz RP. Improvements in outcomes in methadone patients on probation/parole regardless of counseling early in treatment. J Addict Med. 2013 Mar-Apr;7(2):133-8. doi: 10.1097/ADM.0b013e318284a0c1. PubMed PMID: 23455877; PubMed Central PMCID: PMC3618548. |
|  | Gryczysnki J, Schwartz R, O'Grady K, Jaffe J. Dropout from interim methadone and subsequent comprehensive methadone maintenance. Am J Drug Alcohol Abuse. 2009 Dec 15;35(6):394-398. PubMed PMID: 22053122; PubMed Central PMCID: PMC3205350. |
| On-going study, no results published yet | Schwartz RP, Kelly SM, Mitchell SG, Dunlap L, Zarkin GA, Sharma A, O'Grady KE, Jaffe JH. Interim methadone and patient navigation in jail: Rationale and design of a randomized clinical trial. Contemp Clin Trials. 2016 Jul;49:21-8. doi: 10.1016/j.cct.2016.06.002. Epub 2016 Jun 7. Erratum in: Contemp Clin Trials. 2016 Nov;51:97. PubMed PMID: 27282117; PubMed Central PMCID: PMC4969178. |
|  | Chandler RK, Finger MS, Farabee D, Schwartz RP, Condon T, Dunlap LJ, Zarkin GA, McCollister K, McDonald RD, Laska E, Bennett D, Kelly SM, Hillhouse M, Mitchell SG, O'Grady KE, Lee JD. The SOMATICS collaborative: Introduction to a National Institute on Drug Abuse cooperative study of pharmacotherapy for opioid treatment in criminal justice settings. Contemp Clin Trials. 2016 May;48:166-72.  doi: 10.1016/j.cct.2016.05.003. Epub 2016 May 11. PubMed PMID: 27180088; PubMed Central PMCID: PMC5454801. |
|  | NCT03616236. Buprenorphine for Probationers and Parolees: bridging the Gap Into Treatment. Https://Clinicaltrials.Gov/Show/Nct03616236 2018. |
|  | NCT03420313. Interim Buprenorphine Treatment to Bridge Waitlist Delays: stage II Evaluation. Https://Clinicaltrials.Gov/Show/Nct03420313 2018. |
| Congress poster/ presentation/ conference/ comment on other articles | Streck J.M., Ochalek T.A., Hruska B, Pusey J.D., Sigmon S.C. Improvement in psychiatric symptoms during interim buprenorphine treatment. Drug and Alcohol Dependence. Conference: 2016 Annual Meeting of the College on Problems of Drug Dependence, CPDD 2016. United States. 171 (Pp E198), 2017. Date of Publication: 01 Feb 2017. 2017. |
|  | Sigmon S.C., Ochalek T.A., Hruska B et al. Interim buprenorphine treatment for reducing illicit opioid use during treatment delays. Drug and Alcohol Dependence. Conference: 2016 Annual Meeting of the College on Problems of Drug Dependence, CPDD 2016. United States. 171 (Pp E190-E191), 2017. Date of Publication: 01 Feb 2017. 2017. |
|  | Sigmon S.C., Meyer A.C., Hruska B et al. Interim buprenorphine treatment: Leveraging technology to bridge waitlist delays. Drug and Alcohol Dependence. Conference: 2015 Annual Meeting of the College on Problems of Drug Dependence, CPDD 2015. Phoenix, AZ United States. Conference Publication: (Var.Pagings). 156 (Pp E204), 2015. Date of Publication: 01 Nov 2015. 2015. |
|  | Sigmon S. Initial efficacy of interim buprenorphine dosing for reducing illicit drug use and associated risks among waitlisted opioid-dependent adults. Neuropsychopharmacology. Conference: 56th Annual Meeting of the American College of Neuropsychopharmacology, ACNP 2017. United States. 43 (Supplement 1) (Pp S61-S62), 2017. Date of Publication: November 2017. 2017. |
|  | Ochalek T.A., Pusey J.D., Hruska B et al. Within-subject evaluation of interim buprenorphine vs. waitlist on illicit opioid use. Drug and Alcohol Dependence. Conference: 2016 Annual Meeting of the College on Problems of Drug Dependence, CPDD 2016. United States. 171 (Pp E154), 2017. Date of Publication: 01 Feb 2017. 2017. |
|  | Clark N. High dose buprenorphine may be an effective interim treatment for long term heroin users waiting for drug-assisted rehabilitation. Evid Based Ment Health. 2003 Feb;6(1):30. PubMed PMID: 12588836. |
|  | Wilson M, Schwartz RP, O'Grady KE, Highfield D, Jaffe JH. Interim methadone maintenance reduces HIV-risk behaviors. Proceedings of the 69th Annual Scientific Meeting of the College on Problems of Drug Dependence; 2007 June 16-21; Quebec City, Canada 2007. |
|  | Schwartz RP, Kelly SM, O’Grady KE, Jaffe JH. A randomized trial of entry into methadone treatment via interim maintenance: preliminary findings. Proceedings of the 72th Annual Scientific Meeting of the College on Problems of Drug Dependence; 2010 June 12-17; Scottsdale, Arizona. USA 2010; 147. |
|  | Schwartz RP, Jaffe JH, Kelly SM, Gandhi D, O'Grady KE. Entry into methadone, treatment via interim maintenance: 12-month outcomes. Proceedings of the 73rd Annual Scientific Meeting of the College on Problems of Drug Dependence; 2011 June 18-23, Hollywood, Florida 2011; 159, Abstract no: 636. |
|  | Schwartz RP, Highfield DA, BAttjes RJ et al. Interim methadone maintenance: 10 month follow-up. Proceedings of the 67th Annual Scientific Meeting of the College on Problems of Drug Dependence; 2005 June 19-23; Orlando, Florida, USA 2005. |
|  | Schwartz RP, Highfield D, Battjes RJ et al. Interim methadone maintenance: preliminary findings. Proceedings of the 65th Annual Scientific Meeting of the College on Problems of Drug Dependence; 2003 June; Bal Harbour, Florida. USA 2003; 593. |
|  | Kelly SM, Jaffe JH, O'Grady KE, Gandhi D, Schwartz RP. Methadone with or without counseling: impact on HIV-risk behaviors. Proceedings of the 73rd Annual Scientific Meeting of the College on Problems of Drug Dependence; 2011 June 18-23, Hollywood, Florida 2011; 86, Abstract no: 343. |
