# Supplementary material for: Interim opioid agonist treatment for opioid addiction: a systematic review
Source: Harm Reduct J. 2022 Jan 29;19:7. doi: 10.1186/s12954-022-00592-x (PMC8800211; doi:10.1186/s12954-022-00592-x)
Supplement: Supplementary file 4 — Additional file 4. Table of characteristics of included studies. [file 12954_2022_592_MOESM4_ESM.docx]

| **Study ID** | **Yancovitz 1991**  **Main reference**: Yancovitz SR, Des Jarlais DC, Peyser NP, Drew E, Friedmann P, Trigg HL,  Robinson JW. A randomized trial of an interim methadone maintenance clinic. Am J  Public Health. 1991 Sep;81(9):1185-91. PubMed PMID: 1659236; PubMed Central  PMCID: PMC1405649. |
| --- | --- |
| **Objective** | Evaluation of an interim methadone clinic in New York |
| **Methods** | **STUDY DESIGN**  Randomised Trial |
|  | **PARTICIPANTS**  **301 participants** (149 Treatment group, 152 Frequent contact group)  **Inclusion criteria**  Patients from the waiting list of Beth Israel methadone maintenance program. |
|  | **INTERVENTIONS**  **Interim Clinic:** methadone administered by a nurse 5 days a week; Saturday medication and one take-home were provided at another site + AIDS education + free condoms. Minimal counselling on an ad hoc informal basis and other supportive service needs could be addressed only by referral to community agencies. Biweekly urine samples. Results of the urinalysis were known to the treatment staff, but not incorporated into any treatment plan or discussed in formal counseling sessions, as they generally would be in a comprehensive methadone treatment program. The physician could identify medical problems but, except for the most routine of interventions, had to refer patients elsewhere for treatment.  The initial dose of methadone was set by the examining physician based on the examination and drug use history and was typically 20 to 30 mg/day. This initial dose was then increased according to an escalation schedule preset by the physician; generally by 10 mg every third day until a maintenance dosage of approximately 80 mg/day was reached. The nurse was authorized to modify the rate of dose escalation or to stop the escalation process at a stabilization dose other than the scheduled maximum. Dosage adjustment decisions generally reflected subjective perceptions of the patients and instances of missed or vomited medication.  **Control:** waiting list   - First three months, two control groups:   - Frequent control group: biweekly follow-up and urine samples, and free condoms   - Minimal contact control group: not interviewed until the end of participation (data not included in report) - After three months, one control group:   - Frequent contact control group: time spent limited to one month and then switched into experimental group. |
|  | **OUTCOMES**  **Primary**  Change in heroin use  **Secondary**  Changes in cocaine use  Entry into conventional treatment |
| **Notes** | N/A |

# Additional file 4 Table of characteristics of included studies

|  | **Risk of bias**  **Cochrane Risk of Bias Tool*** | **Judgement** | **Support for judgement** |
| --- | --- | --- | --- |
| Selection bias | Random sequence generation | Unclear | **Quote**: “*Subjects were assigned randomly to experimental treatment or control conditions. The random assignment was done by administrative staff at a different location.”* |
|  | Allocation concealment | Low | **Quote**: “*Subjects were assigned randomly to experimental treatment or control conditions. The random assignment was done by administrative staff at a different location.* *The intake interviewers frequently asked to have individuals placed in the methadone treatment group based on data obtained in the intake interview, but, consistent with the protocol, these requests were not honoured.”* |
| Performance bias | Blinding of participants and personnel  (objective and subjective outcomes) | High | **Quote***: “methadone administered by a nurse 5 days per week.”*  **Comment**: Likely high; blinding seems difficult with this design because the experimental group is receiving medication (methadone) and control group is not, therefore either participant and personnel will likely be aware of what group they belong. Nevertheless this is not specifically stated in the report. |
| Detection bias | Blinding of outcome assessment  (objective outcomes) | Unclear | **Quote***: “A follow-up questionnaire, focusing on drug use since the preceding interview, was administered every 2 weeks.”*  **Quote**: “*One research staff member was designated to conduct baseline and biweekly research interviews for the experimental and control subjects.”*  **Comment**: There isn’t any information regarding the blinding of the outcome assessment |
| Detection bias | Blinding of outcome assessment  (subjective outcomes) | Unclear | **Quote***: “A follow-up questionnaire, focusing on drug use since the preceding interview, was administered every 2 weeks.”*  **Quote**: “*One research staff member was designated to conduct baseline and biweekly research interviews for the experimental and control subjects.”*  **Comment**: There isn’t any information regarding the blinding of the outcome assessment |
| Attrition bias | Incomplete outcome data | High | **Quote:** *“Of the 301 subjects initially enrolled, no follow-up data were obtained from 41 subjects (20 from the experimental group, 21 from the control group). Of these 41 subjects, 32 were lost to contact, 8 were admitted to traditional drug treatment, and 1 was incarcerated before follow-up data could be collected.”*  **Comment**: Demographic and drug use history don’t differ significantly between groups at baseline.  Moreover, the study doesn´t report an estimate of the sample size, therefore we don’t know if the drop-out rates were expected or not. |
| Reporting bias | Selective reporting bias | Unclear | **Comment**: Protocol not available therefore we can’t be sure that all pre-specified and expected outcomes of interest are reported |
| Other bias | Other bias | N/A | N/A |

*****[**http://methods.cochrane.org/bias/assessing-risk-bias-included-studies**](http://methods.cochrane.org/bias/assessing-risk-bias-included-studies)

| **Study ID** | **Schwartz 2006**  **Main reference**: Schwartz RP, Highfield DA, Jaffe JH, Brady JV, Butler CB, Rouse CO, Callaman  JM, O’Grady KE, Battjes RJ. A randomized controlled trial of interim methadone maintenance. Arch Gen Psychiatry. 2006 Jan;63(1):102-9. PubMed PMID: 16389204.  **Other references:**  Schwartz RP, Jaffe JH, Highfield DA, Callaman JM, O’Grady KE. A randomized controlled trial of interim methadone maintenance: 10-Month follow-up. Drug  Alcohol Depend. 2007 Jan 5;86(1):30-6. Epub 2006 Jun 21. PubMed PMID: 16793221.  Wilson ME, Schwartz RP, O’Grady KE, Jaffe JH. Impact of interim methadone maintenance on HIV risk behaviors. J Urban Health. 2010 Jul;87(4):586-91. doi:  10.1007/s11524-010-9451-7. PubMed PMID: 20386992; PubMed Central PMCID:  PMC2900571.  Schwartz RP, Jaffe JH, O’Grady KE, Kinlock TW, Gordon MS, Kelly SM, Wilson ME,  Ahmed A. Interim methadone treatment: impact on arrests. Drug Alcohol Depend.  2009 Aug 1;103(3):148-54. doi: 10.1016/j.drugalcdep.2009.03.007. Epub 2009 May  14. PubMed PMID: 19443133; PubMed Central PMCID: PMC2699328. |
| --- | --- |
| **Objective** | Compare the effectiveness of interim methadone maintenance with that of the usual waiting list condition in terms of:   - Facilitating methadone treatment entry - Reducing heroin and cocaine use - Reducing criminal activity - Impact on HIV risk behaviours |
| **Methods** | **STUDY DESIGN**  Two group RCT |
|  | **PARTICIPANTS**  **319 heroin dependent adults** (3:2, 199 Treatment group-IM, 120 Waiting list )  **Inclusion criteria**  Criteria for methadone maintenance (at least 1 year of meeting the DSM-IV criteria for current heroin dependence)  Willing to provide informed consent  No treatment slot was expected to be available in the next 2 weeks.  **Exclusion criteria**  Pregnancy  Acute medical or psychiatric illness. |
|  | **INTERVENTIONS**  **Interim Treatment:** consisting in a brief orientation to the MTP (methadone treatment program) by the program’s emergency counsellor and a physical examination conducted by the program’s medical director. Methadone dosing began the following day at 20 mg, and increased by 5 mg/d, with a target of 80 mg. Participants were able to slow or stop their dose induction by discussion with program nurses, and they were able to exceed the 80-mg target by asking the emergency counselor for a dose increase. Methadone provided under direct observation, participants attended 7 days a week (as the Federal regulation required). 3 consecutive unexcused missed doses resulted in discharge from treatment. Participants assigned to the interim treatment condition were told that they would receive interim treatment without charge for up to 120 days, after which they would be transferred to comprehensive methadone treatment if they were not able to gain entry into a comprehensive MTP before that time.  **Control:** waiting list: no further contact with clinical staff unless their name came up on the waiting list. |
|  | **OUTCOMES**  **Primary**  Entry into comprehensive Methadone Treatment  Self-reported days of heroin use  Self-reported days of cocaine use  Self-reported criminal behaviour  Number of urine drug test results positive for heroin and cocaine  Retention in treatment  **Secondary**  Impact on arrests  HIV risk behaviours |
| **Notes** | N/A |

|  | **Risk of bias**  **Cochrane Risk of Bias Tool*** | **Judgement** | **Support for judgement** |
| --- | --- | --- | --- |
| Selection bias | Random sequence generation | Low | **Quote**: “*The project director (D.A.H.) obtained the random assignment to treatment conditions from a table of random numbers and sealed it in an envelope provided to the research assistant.”* |
|  | Allocation concealment | Low | **Quote**: “*The project director (D.A.H.) obtained the random assignment to treatment conditions from a table of random numbers and sealed it in an envelope provided to the research assistant.”* |
| Performance bias | Blinding of participants and personnel  (objective and subjective outcomes) | High | **Quote***: “.On completion of informed consent and the baseline assessment for an eligible participant, the research interviewer opened the envelope with the condition assignment provided by the project director and informed the participant of his or her assignment to a treatment condition.”*  **Quote**: *“Those participants assigned to the interim treatment condition were told that they would receive interim treatment without charge for up to 120 days, after which they would be transferred to comprehensive methadone treatment if they were not able to gain entry into a comprehensive MTP before that time.”* |
| Detection bias | Blinding of outcome assessment  (objective outcomes) | Low | **Quote***: “Participants were* ***assessed at baseline*** *and when they entered regular methadone treatment, or 120 days from baseline for those who did not enter methadone treatment, by 1 of 2 trained and* ***unblinded research assistants*** *between September 9, 2002, and April 21, 2004.”*  **Quote**: *“****Recent heroin and cocaine use was also measured by a urine drug test; samples were collected at intake and follow-up under observation and analyzed by a certified laboratory using an enzyme-multiplied immunoassay test****.”*  **Comment:** There is no blinding for the outcome assessment as both researchers and participants know the group assignment, nevertheless the measurement and interpretation of objective outcomes (such as drug test, retention in treatment or entry into standard maintenance treatment) is unlikely to be influenced by this. |
| Detection bias | Blinding of outcome assessment  (subjective outcomes) | High | **Quote***: “Participants were* ***assessed at baseline*** *and when they entered regular methadone treatment, or 120 days from baseline for those who did not enter methadone treatment, by 1 of 2 trained and* ***unblinded research assistants*** *between September 9, 2002, and April 21, 2004.”*  **Quote**: “*Self-reported alcohol, heroin, and cocaine use was measured by the Addiction Severity Index (ASI).”*  **Comment:** There is no blinding for the outcome assessment as both researchers and participants know the group assignment, therefore the measurement and interpretation of subjective outcomes (such as self-reported outcomes) is highly likely to be influenced by this. |
| Attrition bias | Incomplete outcome data | High | **Quote**: *“All analyses were conducted on an intent-to-treat basis, using data from the full sample (N=319) described in Table 1. All 319 randomly assigned participants were tracked for follow-up. A total of 302 participants (94.7%) were located for follow- up interviews, and 294 of those located completed interviews*  *(95% of interim and 89% of waiting list participants). The reasons for missing interviews are outlined in the Figure.”*  **Quote**: *“There were no significant differences in baseline characteristics between those participants who were vs those participants who were not unavailable for follow-up, except that participants who missed the follow-up interview had a 79.4% positive rate for cocaine at baseline vs a 60.4% positive rate for those with baseline and follow-up interviews (P=.03).”*  **Comment:** The initial target sample size calculated 360 participants assuming a 5% attrition rate, nevertheless only 334 participants were assessed for eligibility and only 319 were randomised. More over 11 were lost to follow up in the intervention group and 13 for the waiting list group, also 33 participants discontinued the intervention and 0 participants discontinued the waiting list.  Also, despite mentioning that they performed and intention to treat analysis, analysing 199 and 120 patients in each group, they also mention that they exclude 11 patients from analysis in the intervention group and 13 in the waiting list group.  They also mention that because of missing data, sample sizes vary for each outcome. |
| Reporting bias | Selective reporting bias | Unclear | **Comment**: Protocol not available therefore we can’t be sure that all pre-specified and expected outcomes of interest are reported |
| Other bias | Other bias | N/A | N/A |

*****[**http://methods.cochrane.org/bias/assessing-risk-bias-included-studies**](http://methods.cochrane.org/bias/assessing-risk-bias-included-studies)

| **Study ID** | **NCT: NCT02360007**  **Main reference**: Sigmon SC, Ochalek TA, Meyer AC, Hruska B, Heil SH, Badger GJ, Rose G,  Brooklyn JR, Schwartz RP, Moore BA, Higgins ST. Interim Buprenorphine vs. Waiting  List for Opioid Dependence. N Engl J Med. 2016 Dec 22;375(25):2504-2505. doi:  10.1056/NEJMc1610047. PubMed PMID: 28002704; PubMed Central PMCID: PMC5373028..  **Other references:** Streck JM, Ochalek TA, Badger GJ, Sigmon SC. Interim buprenorphine treatment during delays to comprehensive treatment: Changes in psychiatric symptoms. Exp Clin Psychopharmacol. 2018 Aug;26(4):403-409. doi: 10.1037/pha0000199. Epub 2018 Jun 25. PubMed PMID: 29939049; PubMed Central PMCID: PMC6072576 |
| --- | --- |
| **Objective** | To evaluate the efficacy of an interim regimen of buprenorphine for reducing illicit opioid and its impact on psychiatric symptoms. |
| **Methods** | **STUDY DESIGN**  RCT |
|  | **PARTICIPANTS**  **50 opioid dependent adults awaiting agonist maintenance** (25 Interim Buprenorphine Treatment (IBC), 25 Waiting list Control (WLC) )  **Inclusion criteria: ≥**18 years old, in good health, meeting DSM-IV criteria for opioid dependence, that provided an opioid-positive urine and being waitlisted for methadone or buprenorphine treatment.  To minimize disruption due to treatment becoming available during the study, enrolment was limited to those who joined a waitlist in the prior 12 months.  **Exclusion criteria:** having significant and unstable psychiatric or medical illness that could interfere with consent or participation, being pregnant or nursing, being physically dependent on sedative-hypnotics or alcohol. |
|  | **INTERVENTIONS**  **Interim Treatment:** IBT participants will complete buprenorphine (BUP) induction in Week 1 (or longer if required), during which they will attend the clinic daily. Thereafter, during Weeks 2-12 IBT participants will visit the clinic every two weeks to ingest their BUP dose, provide a urine specimen and receive their remaining doses in the Med-O-Wheel.  IBT participants will receive buprenorphine sublingual tablets. Medication will be ordered and managed through our hospital’s investigational pharmacy, which has prepared medications for our prior NIDA grants (e.g., Sigmon et al., 2009, 2013). BUP induction will occur in Week 1 (or longer if required), during which participants will attend the clinic daily. Self-report and observer ratings of withdrawal and agonist effects will be completed at each visit, and urine and breath samples will be collected to ensure no recent use of drugs contraindicated with BUP. Individualized induction will be conducted using a protocolized approach (Johnson et al., 2003; Sigmon et al., 2009, 2013). During Weeks 2-12, participants will visit the clinic once every two weeks to ingest their dose, provide a urine specimen and receive their remaining 13 doses dispensed in the Med-O-Wheel for ingestion at home. They can also return to the clinic between scheduled visits if any concerns arise or if a dose evaluation is needed. At the end of the study, participants will be offered the 4-week BUP taper that was shown in our prior RCT to produce favourable outcomes (Sigmon et al., 2013) or, if a treatment slot has become available at their desired clinic, we will work with clinic staff to ensure a smooth transition to that program. If the participant elects to receive the 4-week buprenorphine taper, we will use the same procedures used in the aforementioned study (CHRMS 06-213/Sigmon et al., 2013), which will include daily visits, daily dosing of gradually-decreasing BUP doses and provision of non-opioid ancillary medications (e.g., clonidine, hydroxyzine) as needed for managing any opioid withdrawal symptoms.  **Control:** WLC participants will remain on the waitlist for their treatment of choice. Participants assigned to the WLC will remain on the waiting list for their treatment of choice. They will visit the clinic to complete follow-up assessments and provide staff-observed urines according to the same schedule as IBT participants (Weeks 4, 8, 12, 18, 24). WLC participants who have not entered agonist treatment by Week 12 (which we anticipate to be the majority) will be offered the opportunity to receive IBT for an additional 12-week period as described above. This will permit an additional within-subject opportunity to qualitatively evaluate the size of IBT effects, as well as being an ethical strength by providing WLC participants the opportunity to receive active treatment.  Participants in both conditions will complete follow-up assessments and provide a urine specimen at 4, 8, 12, 18 and 24 weeks after trial entry. WLC participants who have not entered treatment by Week 12 will be offered IBT at that time, providing an additional within-subject evaluation of IBT effects. Thus the overall possible study duration may vary between 12 - 28 weeks. |
|  | **OUTCOMES**  **Primary**  illicit opioid abstinence  **Secondary**  Psychosocial functioning (i.e., ASI subscale scores)  Intravenous drug use  ASI scores  Adherence to treatment regimen  Patient satisfaction  Change in psychiatric symptoms (secondary analysis) |
| **Notes** | N/A |

|  | **Risk of bias**  **Cochrane Risk of Bias Tool*** | **Judgement** | **Support for judgement** |
| --- | --- | --- | --- |
| Selection bias | Random sequence generation | Low | **Comment**: The study protocol reports that a minimum likelihood allocation was use to ensure balance between study arms. |
|  | Allocation concealment | Unclear | **Comment**: The researchers did not provide details on the efforts made to conceal the random allocation |
| Performance bias | Blinding of participants and personnel  (objective and subjective outcomes) | High | **Comment**: The trial was registered as open label, according the clinicaltrials.gov record. |
| Detection bias | Blinding of outcome assessment  (objective outcomes) | Unclear | **Comment**: There is no information in the protocol nor the article about the blinding of outcome assessment |
| Detection bias | Blinding of outcome assessment  (subjective outcomes) | Unclear | **Comment**: There is no information in the protocol nor the article about the blinding of outcome assessment |
| Attrition bias | Incomplete outcome data | High | **Quote**: (from the protocol) *“The proposed sample of 70 subjects is based on having sufficient power for detecting a group difference on the percent of participants negative for illicit opioids at Week 12. Power is estimated to be 90% using α=.05 if the true abstinence rates are 60% vs. 20% for the IBT and WLC groups, respectively. These estimates are based on the IMT study by Schwartz et al. (2006), with slightly higher abstinence expected in our IBT condition as it is more intensive than the intervention used in that trial.”*  **Comment**: The final sample was 50, and it isn’t clear why there are 20 participants less than stated initially.  Results are also given in only in percentage, without stating the number of patients nor mentioning if any patients have been lost during the study follow up. |
| Reporting bias | Selective reporting bias | High | **Comment**: Protocol is available and the trial was also registered at clinicaltrials.gov. Primary outcomes specified in the protocol are illicit opioid abstinence and psychosocial functioning but the latter is reported as secondary outcome in the report. Furthermore, there is one secondary outcome specified in the protocol (acceptability) that is not reported. |
| Other bias | Other bias | N/A | N/A |

*****[**http://methods.cochrane.org/bias/assessing-risk-bias-included-studies**](http://methods.cochrane.org/bias/assessing-risk-bias-included-studies)

| **Study ID** | **NCT: NCT00712036**  **Main reference**: Schwartz RP, Kelly SM, O’Grady KE, Gandhi D, Jaffe JH. Randomized trial of standard methadone treatment compared to initiating methadone without counseling: 12-month findings. Addiction. 2012 May;107(5):943-52. doi: 10.1111/j.1360-0443.2011.03700.x. Epub 2012 Feb 11. PubMed PMID: 22029398; PubMed Central PMCID: PMC3319854.  **Other references:**  Schwartz RP, Kelly SM, O’Grady KE, Gandhi D, Jaffe JH. Interim methadone treatment compared to standard methadone treatment: 4-month findings. J Subst Abuse Treat. 2011 Jul;41(1):21-9. doi: 10.1016/j.jsat.2011.01.008. Epub 2011 Feb 24. PubMed PMID: 21353445; PubMed Central PMCID: PMC3110526.  Schwartz RP, Alexandre PK, Kelly SM, O’Grady KE, Gryczynski J, Jaffe JH. Interim versus standard methadone treatment: a benefit-cost analysis. J Subst Abuse Treat. 2014 Mar;46(3):306-14. doi: 10.1016/j.jsat.2013.10.003. Epub 2013 Oct 14. PubMed PMID: 24239030; PubMed Central PMCID: PMC3947051.  Kelly SM, Schwartz RP, Oʼgrady KE, Gandhi D, Jaffe JH. Impact of methadone with versus without drug abuse counseling on HIV risk: 4- and 12-month findings from a clinical trial. J Addict Med. 2012 Jun;6(2):145-52. doi: 10.1097/ADM.0b013e31823ae556. PubMed PMID: 22134175; PubMed Central PMCID: PMC3322294. |
| --- | --- |
| **Objective** | To determine the relative effectiveness of 4 months and 12-months of Interim Methadone (IM; supervised methadone with emergency counseling only for the first 4 months of treatment), Standard Methadone treatment (SM; with routine counseling) and Restored Methadone treatment (RM: routine counseling with smaller caseloads) and to conduct a benefit-cost analysis. |
| **Methods** | **STUDY DESIGN**  RCT |
|  | **PARTICIPANTS**  **230 heroin dependent adults** (99 IM participants, 104 SM participants, 27 RM participants)  **Inclusion criteria:**  (1) be at least 18 years old;  (2) be willing to provide informed consent for study participation; and  (3) meet criteria for admission to an MTP (at least one year of meeting DSM-IV criteria for opioid dependence).  4 unable to gain admission to methadone program within 14 days  **Exclusion criteria:** being pregnant or having an acute medical or psychiatric illness that required immediate treatment. |
|  | **INTERVENTIONS**  **Interim Treatment (IM):** Methadone maintenance for up to 4 months with emergency counseling only for individuals on program waiting lists. Treatment was provided following federal regulations (42 CFR 8.12), which require: 1) daily methadone administration (i.e., no take-home doses); 2) that IM cannot exceed 120 days; and, 3) a minimum of three drug tests. Although no regularly scheduled counseling is available in IM, there was a counselor available to deal with participant crises.  **Control:**  **Standard methadone treatment (SM):** Methadone treatment with counseling as usual. Treatment was provided in keeping with federal and state regulations, current accrediting standards, and the MTPs usual practices, which permitted take-home doses contingent upon tenure and progress in treatment (e.g., negative drug tests), required regularly scheduled counseling, treatment planning, other psychosocial treatment as needed, and more frequent drug testing than in IM. In keeping with the usual practice of the participating MTPs, participants assigned to SM were expected to attend weekly group and/or individual counseling sessions, although the frequency of counseling could vary with the needs of the participant. Payment for both treatments was based on Maryland’s sliding fee scale. Indigent participants at one site had their fee waived and at the second site were asked to pay $2.00 per week.  **Restored Methadone treatment (RM):** Methadone Treatment with counseling provided by a clinician with a lower caseload than counseling as usual. Provided participants with all the services offered in SM. For this Condition, the second site was given additional financial support and the clinic director agreed to provide one of that MTP’s best counselors to provide counseling. That counselor carried a reduced case load not to exceed 25 patients. Such a caseload represented half of the maximum caseload permitted by the Maryland Opioid Treatment Program regulations (Code of Maryland Regulations: 10.47.02.11). Other than the lower case load, the counselor was instructed to see the participants as frequently as the participants wanted and the counselor deemed appropriate.  Methadone dosing in all conditions was provided in a similar manner, usually beginning at around 20 mg daily with gradual daily or every other day increases to an initial target of between 60 and 80 mg. |
|  | **OUTCOMES**  **Primary**  Opiate positive drug test  **Secondary**  Cocaine positive drug test  Retention in treatment  Self-reported heroin use  Self-reported cocaine use  Self-reported criminal behaviour and arrests  Benefit-cost evaluation  HIV risk |
| **Notes** | N/A |

|  | **Risk of bias**  **Cochrane Risk of Bias Tool*** | **Judgement** | **Support for judgement** |
| --- | --- | --- | --- |
| Selection bias | Random sequence generation | Low | **Quote**: *“After they provided informed consent and completed their baseline assessment and their MTP admission procedures, participants were block randomized to study condition using a computer-generated procedure.”* |
|  | Allocation concealment | Low | **Quote**: *“At study onset, the project manager used this list to create a series of numbered cards with the study ID and assigned condition, inserting them into numbered, opaque envelopes.”*  **Quote**: *“Following completion of the informed consent process, baseline assessment procedures, and the physical performed at the program, the research assistant opened a sealed, numbered, and opaque envelope provided by the study’s project director to assign the participants’ study condition”* |
| Performance bias | Blinding of participants and personnel  (objective and subjective outcomes) | High | **Quote**: *“MTP staff was not blind to condition post-randomization assignment.”* |
| Detection bias | Blinding of outcome assessment  (objective outcomes) | Low | **Quote**: *“Participants were assessed at baseline by research assistants (RAs) […] in an unblinded fashion at 4 and 12 months post-baseline”*  **Comment**: Follow-up assessments were not blinded but it is unlikely that the measurements and interpretation of objective outcomes (such as drug tests) were biased. |
| Detection bias | Blinding of outcome assessment  (subjective outcomes) | High | **Quote**: *“Participants were assessed at baseline by research assistants (RAs) […] in an unblinded fashion at 4 and 12 months post-baseline”*  **Comment**: Follow-up assessments were not blinded therefore it is highly likely that the measurements and interpretation of subjective outcomes (such as self-reported outcomes) were biased. |
| Attrition bias | Incomplete outcome data | Low | **Comment**: 244 people randomised- IM 108, SM 107, RM 29.  230 people received some kind of intervention- IM 99 (9 excluded for psychiatric illness or not being admitted by MTP or not being heroin-addicted), SM 104 (3 excluded for not being admitted by MTP), RM 27 (2 excluded for having a family member in the same condition or being enrolled in another study).  216 people with follow up at 4 months and analysed – IM 97 (1 in jail, 1 not located), SM 93 (6 in jail, 4 not located, 1 quit), RM 26 (1 quit)  210 people with follow up at 12 months and analysed – IM 93 (2 in jail, 2 dead, 2 not located), SM 92 (4 in jail, 1 refusal, 7 not located), RM 25 (1 in jail, 1 refusal)  **Quote**: *“Data were analyzed using the sample of 230 participants who were randomly assigned to condition and received at least one dose of methadone.”* |
| Reporting bias | Selective reporting bias | Low | **Comment** The trial was registered at clinicaltrials.gov and main results in primary and secondary outcomes (drug use outcomes, retention in treatment, crime outcomes, HIV risk and cost-benefit) were reported, as well as other outcomes such as serious adverse events and other treatment outcomes |
| Other bias | Other bias | N/A | N/A |

*****[**http://methods.cochrane.org/bias/assessing-risk-bias-included-studies**](http://methods.cochrane.org/bias/assessing-risk-bias-included-studies)

| **Study ID** | **Krook 2002**  **Main reference**: Krook AL, Brørs O, Dahlberg J, Grouff K, Magnus P, Røysamb E, Waal H. A placebo-controlled study of high dose buprenorphine in opiate dependents waiting for medication-assisted rehabilitation in Oslo, Norway. Addiction. 2002 May; 97(5):533-42. PubMed PMID: 12033654. |
| --- | --- |
| **Objective** | To evaluate whether buprenorphine, even without additional control and psychosocial treatment and support, alleviates the problems faced by patients waiting for medication assisted rehabilitation (MAR). |
| **Methods** | **STUDY DESIGN**  RCT (randomized double blind study) |
|  | **PARTICIPANTS**  **106 opioid dependent adults on the waiting list for MARIO (55 buprenorphine, 51 placebo)**  **Inclusion criteria:**  being at least 25 years old;  having more than 10 years of opioid dependence;  have a written plan for the rehabilitation process:  traditional drug-free treatment should already have been attempted;  to have a current opioid dependence  **Exclusion criteria:** Serious illness and pregnancy |
|  | **INTERVENTIONS**  **Buprenorphine:** buprenorphine without additional rehabilitation or support**.** 4 mg Subutex on the first day, increase to 16mg/d on the following 8 days. At the 12^th^ week, dosage was scaled down to 4mg/d. This was necessary because both groups would have the opportunity to continue with buprenorphine after the study period and at that time we did not know to which group the patients had been assigned.  **Control:** Placebo without additional rehabilitation or support. 4mg of Subutex on the first day and then the dose was decreased to 0 in 9 days and thereafter replaced by placebo.  On both groups, every week (Monday–Saturday) patients were given the tablets sublingually by a nurse/counsellor in groups of 10. They had to come at the appointed time. Two staff members observed the patients until the tablets were absorbed, approximately 10 minutes. On Saturday the patients received a double dose. No take-home doses were allowed.  Intake of Subutex was consecutively registered. If a patient came to the appointment time obviously intoxicated, the medication for the corresponding day was withdrawn. For patients who missed 4 consecutive days of dosing, further medication was withdrawn and they were considered non-completers. We considered it too risky to give a patient 16 mg buprenorphine if he or she had been without medication for more than 4 days. |
|  | **OUTCOMES**  **Primary**  Retention (is the patient still in the project?) and compliance (how many of the total number of doses had been taken?)  **Secondary**  Subjective wellbeing (visual analogue scale: 10-very bad; 0-very well)  Life satisfaction (Temporal Satisfaction with Life scale)  Mental health (symptom checklist-SCL5)  Self-reported drug use (visual analogue scale: 10-Daily heavy drug abuse: 0-Drug free)  Adverse effects |
| **Notes** | N/A |

|  | **Risk of bias**  **Cochrane Risk of Bias Tool*** | **Judgement** | **Support for judgement** |
| --- | --- | --- | --- |
| Selection bias | Random sequence generation | Low | **Quote**: *“The randomization code was kept in the pharmacy and was available only to the pharmacy staff.”* |
|  | Allocation concealment | Low | **Quote**: *“The randomization code was kept in the pharmacy and was available only to the pharmacy staff.”* |
| Performance bias | Blinding of participants and personnel  (objective and subjective outcomes) | Low | **Quote***: “At the 12^th^ week of the period the dosage was scaled down to 4 mg/day. This was necessary because both groups would have the opportunity to continue with buprenorphine after the study period and at that time we did not know to which group the patients had been assigned.”*  **Quote**: *“The randomization code was revealed when all patients had finished the 12-week period.”* |
| Detection bias | Blinding of outcome assessment  (objective outcomes) | Low | **Quote**: *“At the 12^th^ week of the period the dosage was scaled down to 4 mg/day. This was necessary because both groups would have the opportunity to continue with buprenorphine after the study period and at that time we did not know to which group the patients had been assigned.”*  ***Quote****: “The randomization code was revealed when all patients had finished the 12-week period.”* |
| Detection bias | Blinding of outcome assessment  (subjective outcomes) | Low | **Quote**: *“At the 12^th^ week of the period the dosage was scaled down to 4 mg/day. This was necessary because both groups would have the opportunity to continue with buprenorphine after the study period and at that time we did not know to which group the patients had been assigned.”*  ***Quote****: “The randomization code was revealed when all patients had finished the 12-week period.”* |

| Attrition bias | Incomplete outcome data | Unclear | **Quote**: “*Seven non-completers (two males and five females) were not available for the follow-up evaluation, four in the buprenorphine group and three in the placebo group. All of them dropped out during the first week due to missing 4 consecutive days of medication.*”  **Quote**: “*The estimate of which treatment was given to the 78 patients who fulfilled ≥ 10 days is reported in Table 2.*”  **Quote**: “*The most common reason for dropping out before 12 weeks was missing 4 consecutive days, 44/50 (88%) in the placebo group and 33/39 (85%) in the buprenorphine group. The others discontinued for other reasons, five (10%) in the placebo group and four (10%) in the buprenorphine group. Six did not finish because they thought they were taking the placebo (one did not), three dropped out because they wanted to continue drug abuse for one more period (all in the Subutex group) and one patient in the placebo group discontinued treatment because of hospitalization for a collum femoris fracture.*  *With regard to the seven patients who dropped out during the initial phase and who did not return for evaluation after 12 weeks, there were no significant differences between this group and the remaining participants (n = 99) […]*”  **Comment**: The report doesn’t include a sample size estimate and the description of the drop out rates doesn’t’ allow us to differentiate between those that are attributable to the intervention and those that aren’t. |
| --- | --- | --- | --- |
| Reporting bias | Selective reporting bias | Unclear | **Comment**: Protocol not available therefore we can’t be sure that all pre-specified and expected outcomes of interest are reported. |
| Other bias | Other bias | N/A | N/A |

*****[**http://methods.cochrane.org/bias/assessing-risk-bias-included-studies**](http://methods.cochrane.org/bias/assessing-risk-bias-included-studies)

| **Study ID** | **Friedmann 1994**  **Main reference**: Friedman P, Des Jarlais DC, Peyser NP, Nichols SE, Drew E, Newman RG.  Retention of patients who entered methadone maintenance via an interim methadone  clinic. J Psychoactive Drugs. 1994 Apr-Jun;26(2):217-21. PubMed PMID: 7931866. |
| --- | --- |
| **Objective** | To compare the retention of patients first admitted to an interim methadone clinic versus that of patients admitted directly to a comprehensive methadone clinic ad to determine whether admission and an initial period of limited services in the interim clinic had an adverse effect on retention. |
| **Methods** | **STUDY DESIGN**  Retrospective cohort study |
|  | **DATA SOURCE**  Computerized data collected as a matter of routine for all of Beth Israel’s methadone clinics, both for clinical and administrative purposes. Subjects were selected for this analysis on the basis of the year of admission, the clinic to which they were admitted, and the geographic area where the clinic was located. |
|  | **PARTICIPANTS**  All **314** **consecutive admissions** to Beth Israel’s Interim Clinic were compared to all **663** **consecutive admissions** to Beth Israel’s traditional methadone clinics (during the years 1990 and 1991).  **Inclusion criteria**: not clearly described |
|  | **INTERVENTIONS**  **Interim methadone treatment:** a physical examination on admission was performed by a physician, methadone was administered by a registered nurse six days per week with one take-home dose for Sunday, and education about AIDS-risk reduction was provided. Only crisis counseling services were provided, and the focus of the Interim Clinic counselors was otherwise limited to AIDS-risk reduction. The clinic had a capacity of 150 patients, thus providing a ratio of 1counselor to 75 patients. Interim methadone treatment did not include the treatment planning and regular counseling required in a traditional methadone clinic .  **Control:** The comparison group includes all consecutive 1990 and 1991 admissions to Beth Israel’s **traditional methadone clinics** located in the same immediate area as the Interim Clinic. If a patient was admitted more than once during the period considered for this analysis, only the first admission was selected. |
|  | **OUTCOMES**  **Primary**  Retention rates in treatment |
| **Notes** | N/A |

|  | **ROBINS-I**** | **Judgement** | **Support for judgement** |
| --- | --- | --- | --- |
| Selection bias | Bias due to confounding | Serious | We suspect there is a risk of bias in this domain because the study only controlled for one of the confounding variables (other substance use) that were defined a priori for the review protocol. Previous participation in methadone treatment is also a potential confounding variable and this was not controlled. |
|  | Bias in selection of participants | Serious | We suspect a serious risk of bias because, taking into account that the main intervention (Interim Clinic) was also affected by a waiting list and therefore admission into interim was not immediate, the decision of entering into the interim treatment when a slot became available or remaining on the waiting list for an opening into the standard maintenance treatment was left at the participants discretion. |
| Information bias | Bias in classification of intervention | Low | Patients are classified and analysed according to the intervention that they were first admitted to, either interim treatment or traditional methadone treatment. |
| Performance bias | Bias due to deviation from intended intervention | Serious | Likely, there were deviations from the way to deliver the protocol at the interim clinic, as many participants also entered into a waiting list because the clinic “was operating at capacity”. |
| Attrition bias | Bias due to missing outcome data | Low | It is a retrospective analysis which included all consecutive admissions to Interim Clinic and to traditional methadone clinics during 1990 and 1991, and no deviations from the management pathway are reported. |
| Measurement bias | Bias in measurement of outcomes | Low | Unlikely bias in the measurement of the outcome of interest (retention) |
| Reporting bias | Bias in selection of the reported result | Low | Unlikely, as only outcome has been reported |

** Sterne JA, BMJ. 2016;355:i4919. doi: 10.1136/bmj.i4919
